# A Novel Macro-Micro Approach for Swimming Analysis in Main Swimming Techniques Using IMU Sensors

**DOI:** 10.3389/fbioe.2020.597738

**Published:** 2021-01-14

**Authors:** Mahdi Hamidi Rad, Vincent Gremeaux, Farzin Dadashi, Kamiar Aminian

**Affiliations:** ^1^Laboratory of Movement Analysis and Measurement, École Polytechnique Fédérale de Lausanne, Lausanne, Switzerland; ^2^Institute of Sport Sciences, University of Lausanne, Lausanne, Switzerland; ^3^Sport Medicine Unit, Division of Physical Medicine and Rehabilitation, Swiss Olympic Medical Center, Lausanne University Hospital, Lausanne, Switzerland; ^4^Gait Up S.A., Lausanne, Switzerland

**Keywords:** sports biomechanics, wearable sensor, swimming, macro-micro analysis, lap segmentation

## Abstract

Inertial measurement units (IMU) are proven as efficient tools for swimming analysis by overcoming the limits of video-based systems application in aquatic environments. However, coaches still believe in the lack of a reliable and easy-to-use analysis system for swimming. To provide a broad view of swimmers' performance, this paper describes a new macro-micro analysis approach, comprehensive enough to cover a full training session, regardless of the swimming technique. Seventeen national level swimmers (5 females, 12 males, 19.6 ± 2.1 yrs) were equipped with six IMUs and asked to swim 4 × 50 m trials in each swimming technique (i.e., frontcrawl, breaststroke, butterfly, and backstroke) in a 25 m pool, in front of five 2-D cameras (four under water and one over water) for validation. The proposed approach detects swimming bouts, laps, and swimming technique in macro level and swimming phases in micro level on all sensor locations for comparison. Swimming phases are the phases swimmers pass from wall to wall (wall push-off, glide, strokes preparation, swimming, and turn) and micro analysis detects the beginning of each phase. For macro analysis, an overall accuracy range of 0.83–0.98, 0.80–1.00, and 0.83–0.99 were achieved, respectively, for swimming bouts detection, laps detection and swimming technique identification on selected sensor locations, the highest being achieved with sacrum. For micro analysis, we obtained the lowest error mean and standard deviation on sacrum for the beginning of wall-push off, glide and turn (−20 ± 89 ms, 4 ± 100 ms, 23 ± 97 ms, respectively), on shank for the beginning of strokes preparation (0 ± 88 ms) and on wrist for the beginning of swimming (−42 ± 72 ms). Comparing the swimming techniques, sacrum sensor achieves the smallest range of error mean and standard deviation during micro analysis. By using the same macro-micro approach across different swimming techniques, this study shows its efficiency to detect the main events and phases of a training session. Moreover, comparing the results of both macro and micro analyses, sacrum has achieved relatively higher amounts of accuracy and lower mean and standard deviation of error in all swimming techniques.

## Introduction

As a highly competitive sport, swimming is one of the most popular events with world-class athletes, aiming to optimize their performance. Among the coach's principal duties are monitoring the swimmers permanently, evaluating their performance and providing feedback for their improvement (Bompa and Buzzichelli, 2018; Marinho et al., 2020). To help coaches with these tasks, research community has studied swimming from various perspectives such as physiology (Pendergast et al., 1980; Lavoie and Montpetit, 1986; Zamparo et al., 2005), motor control (Seifert et al., 2011a; Morais et al., 2020), and biomechanics (Payton and Bartlett, 1995; Morais et al., 2012). Although all these aspects have their own significance, studies show the dominance of biomechanical factors over the other aspects (Figueiredo et al., 2013). Moreover, swimming coaches also consider biomechanics the most critical area of improvement for swimmers (Mooney et al., 2016a).

Using video-based systems is a common tool for motion analysis, which is still considered as the most accurate method and gold standard (Mooney et al., 2015; Seifert et al., 2015). However, as a result of its limitations in aquatic environments (Callaway et al., 2010), the number of studies on swimming with inertial measurement units (IMUs) has been increased (Guignard et al., 2017). There is a multitude of research on measuring the swimming kinematic parameters using IMUs in different swimming phases, such as start (Stamm et al., 2013a; Vantorre et al., 2014), swimming (Ohgi et al., 2003; Davey et al., 2008), or turn (Slawson et al., 2012; Nicol et al., 2018). To evaluate the swimmer's performance, many studies focused on extracting specific parameters such as stroke rate (Siirtola et al., 2011; Beanland et al., 2014), distance per stroke (Bächlin et al., 2008), velocity (Wright and Stager, 2013; Dadashi et al., 2015), lower limbs actions rate (Fulton et al., 2009), or body coordination (Osborough et al., 2010; Silva et al., 2015).

The general approach of most studies is limited to a specific swimming technique or phase. As the most prevalent swimming technique, frontcrawl has been more investigated in the literature (Mooney et al., 2016b) and development of swimming technique specific algorithms is proposed as a future application for IMUs (Magalhaes et al., 2015). Swimming phases are the phases swimmers pass from wall to wall (wall push-off, glide, strokes preparation, swimming, and turn). Among different phases, swimming phase has been noticed the most, while start or turn have not captured enough attention. It is well-established that these phases are of utmost importance for coaches (Mooney et al., 2016b). Another downside is focusing only on a small number of swimmers, lacking variety of technique among subjects (Slawson et al., 2012; Hagem et al., 2013; Seifert et al., 2014).

Using the least number of IMUs is another challenge for a wearable analysis system, as they induce drag unlike video-based systems. By reducing the number of sensors and providing adequate fixation or integrating the wearable sensor into the suit, goggles or watch, swimmers face less drag. Only one study performed a qualitative comparison for possibility of direct or indirect extraction of kinematic parameters with IMU on lower and upper limbs (Pansiot et al., 2010).

As a result, a comprehensive study over different swimming techniques and swimming phases with IMUs on various sensor locations during a training session is necessary to provide a complete view over swimmer's performance from macro level to micro level. All four main swimming techniques i.e., front crawl, breaststroke, butterfly, and backstroke can be decomposed into different locomotion phases from wall to wall. There is an analogy between swimming and gait analysis in terms of the way one can narrow down from a big picture to the detailed parameters, also known as macro-micro approach (Lord et al., 2013). Using body-worn sensors, such as accelerometers, this approach first detects the amount and variability of ambulatory activity (lying, sitting or standing, and gait) as macro level and then continues to gait phases and spatiotemporal parameters as micro level. Likewise in swimming analysis, detecting the amount of swimming (swimming bouts and laps) in different swimming techniques in each lap constitutes the macro level, while the micro level targets detecting the swimming phases in each lap and finally extracting parameters within each swimming phase.

**Figure 1:**
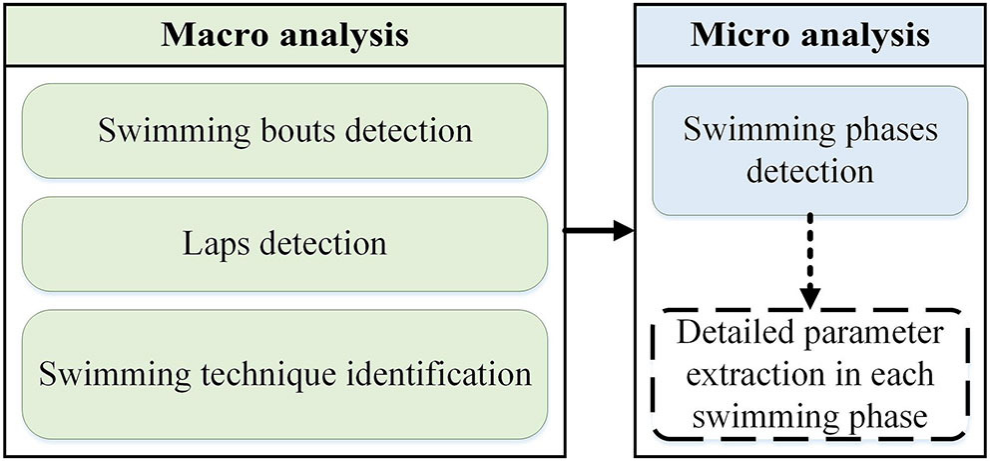
Macro-micro analysis approach diagram to show the scope of this study.

Following this approach, the main objective of this study was to design an IMU-based wearable system for swimming analysis during training sessions including four main swimming techniques. As illustrated in Figure 1, the approach was macro-micro, where swimming bouts, laps, and techniques were detected in macro level, and swimming phases within each lap are identified in micro level. More detailed parameter extraction in each phase (e.g., detecting stroke cycle sub-phases) is the next step of micro analysis, which is out of the scope of this study (Figure 1). This approach aims to provide a thorough view over the swimmer's performance to the coach during each training session.

We hypothesized that changes in motion and posture alter the kinematic profile of wrists, sacrum, head, and shanks motion, which could be recognized by adequate IMU-based algorithms to detect swimming bouts, laps, swimming technique, and subsequently swimming phases. The accuracy and precision of detection algorithms for each sensor location are estimated and compared in order to find the most suitable location for monitoring swimmers' training with this approach. All the abbreviations used in this study are explained in a table of glossary in Supplementary Table 4.

## Materials and Methods

### Measurement Setup

Seventeen national level swimmers (with attributes listed in Table 1) were asked to perform four 50-m trials in each swimming technique in a 25-m indoor pool, with 80% of their best speed. Since swimming analysis during training sessions is the main goal of this research, 80% is considered as a moderate pace close to what used during the training sessions, allowing the balance between speed and motion accuracy (Schmidt and Lee, 2019). The moderate pace helps the swimmers to keep efficient performance while avoiding fatigue in a long training session. Moreover, wearable sensors induce more drag on swimmer's body, specifically in high pace and it is necessary to compensate for this effect by reducing the pace (Magalhaes et al., 2015; Guignard et al., 2017). The trials were separated with a short break, leading to several swimming bouts and the total duration of the measurement was 1 h per swimmer. During the test, the coach was observing and evaluating the pace qualitatively, and asked the swimmers to correct it in case of fast or slow pace. The swimmers are selected from national swimming clubs and practice swimming more than five times per week for competitions. Each swimmer was informed of the procedure and gave their written consent prior to participation. This study was approved by the EPFL human research ethics committee (HREC, No: 050/2018).

**Table 1:** Statistics of the measurement population.

**Male**	**Female**	**Age (yrs)**	**Height (cm)**	**Weight (kg)**	**Record_50m_ (s)**		**FINA_50m_**
12	5	19.6 ± 2.1	179.5 ± 6.7	74.5 ± 7.1	Front crawl	24.56 ± 1.26	725 ± 53
					Breaststroke	32.13 ± 1.52	631 ± 42
					Butterfly	26.86 ± 1.68	652 ± 83
					Backstroke	28.63 ± 1.41	612 ± 95

*All variables are presented as mean ± standard deviation. **Record**_**50m**_ and **FINA**_**50m**_ are the average and standard deviation of 50m record and FINA points (for 2019) of the swimmers separately for each swimming technique*.

**Figure 2:**
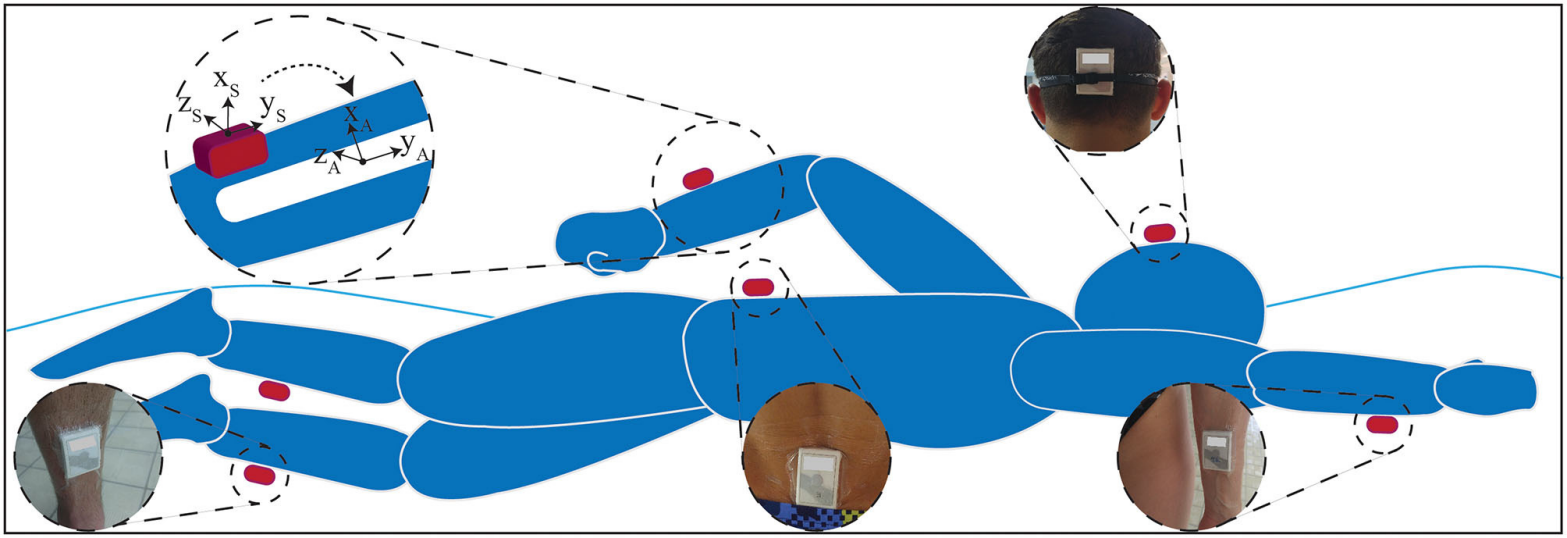
IMU-based measurement setup. Six IMUs were attached to shanks, wrists, sacrum and head using waterproof tapes. During functional calibration, for each segment, the data will be transformed from sensor frame (**xyz**_**S**_) to anatomical frame (**xyz**_**A**_).

A wearable measurement system including six IMUs (Physilog® IV, GaitUp, CH) was used. IMUs were attached to right and left shanks (R/LS), right and left wrists (R/LW), sacrum (SA), and head (HE) using waterproof bands (Tegaderm, 3M Co., USA). The swimmers were asked to wear two swimming caps to keep the head sensor as fixed as possible on the back of their head. The rest of the sensors were taped directly on swimmer's skin. Each unit contained a 3D gyroscope (± 2,000 °/s) and a 3D accelerometer (± 16 g), with a sampling rate of 500 Hz (Figure 2).

**Figure 3:**
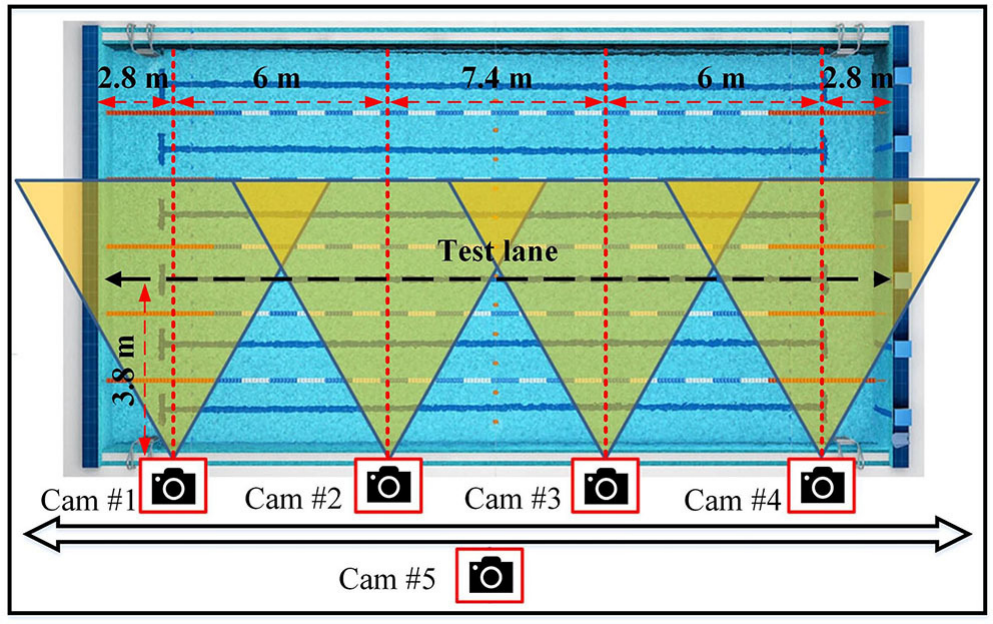
For validation system, four cameras (Cam#1–Cam#4) were distributed along the pool in the same depth (0.5 m) underwater and one camera (Cam#5) moved with the swimmer in land to capture the events over water.

Five 2-D cameras (GoPro Hero 7 Black, GoPro Inc., US) were used for validation, four of them under water (attached to the pool wall, distributed along the length of the pool) to capture all lap events and one camera over water moving with the swimmer (Figure 3), all capturing with a rate of 60 Hz. A push-button, which was used to start the data acquisition by IMUs, also provided a flashlight in front of the cameras to synchronize the two systems. This procedure is done at the beginning and end of each measurement to make sure that the systems remained synched through the measurement.

To make the IMU data independent of sensor exact placement on swimmers' body, a functional calibration was performed after sensors installation. As a result of this calibration, the data will represents the true motion of the limb, regardless of the exact sensor location and the difference of sensor placement on different swimmers or limbs does not affect the data. The purpose of this calibration is to find the transformation matrix that matches the sensor frame (*x*_*s*_, *y*_*s*_, *z*_*s*_)_*i*_ of each sensor i (i = 1,…,6) to the corresponding body segment anatomical frame (*x*_*A*_, *y*_*A*_, *z*_*A*_)_*i*_ (Figure 2). The procedure of functional calibration is explained in Dadashi et al. (2014), which includes simple movements (upright standing, squats, and arm rotation) in land. After this calibration, each sensor coordinate system has its axis y along the longitudinal axis of the limb directed upward (y), x axis along the anterior-posterior axis pointing forward (x), and z axis along the mediolateral axis (z), pointing to the right direction (Figure 2). The trunk pitching and rolling motion while swimming are defined as its rotation around body medial-lateral and inferior-superior axes, respectively.

### Analysis Approach

During a training session, there are several swimming bouts in different swimming techniques (front crawl, breaststroke, butterfly, backstroke), each one consisting of one or more laps. Within each lap, from one pool wall to the other, swimmers pass five main phases: wall push-off (*Push*), glide (*Glid*), strokes preparation (*StPr*), swimming (*Swim*), and turn (*Turn*).

1. Wall push-off Phase starts on the frame with forward motion of swimmer's trunk and finishes upon swimmer's feet leaving the wall (Slawson et al., 2010; Stamm et al., 2013b). This phase is the same for all swimming techniques except it happens in supine posture during backstroke.
2. Glide phase continues as long as swimmer's body glides under water without upper or lower limb movement. This phase ends with butterfly lower limbs action (for front crawl, butterfly, and backstroke) or one upper limbs cycle and then a lower limb action under water (for breaststroke) (Stamm, 2013; Vantorre et al., 2014). Although it is allowed to do one butterfly lower limbs action for breaststroke, the swimmers were trained to follow the traditional method.
3. Strokes preparation is the phase after glide, which continues up to the first upper limbs cycle (Silveira et al., 2011; Vantorre et al., 2014).
4. Swimming phase is usually the longest phase, which lasts as long as the swimmer performs upper limbs cycles. During tumble turn, swimming phase ends with the last upper limbs cycle and head downward motion for rolling, while during simple turn, it finishes by touching the wall (Pereira et al., 2015; Mooney et al., 2016b).
5. Turn phase happens after swimming phase and ends on the frame of the next wall push-off phase start (Le Sage et al., 2010; Vannozzi et al., 2010).

**Figure 4:**
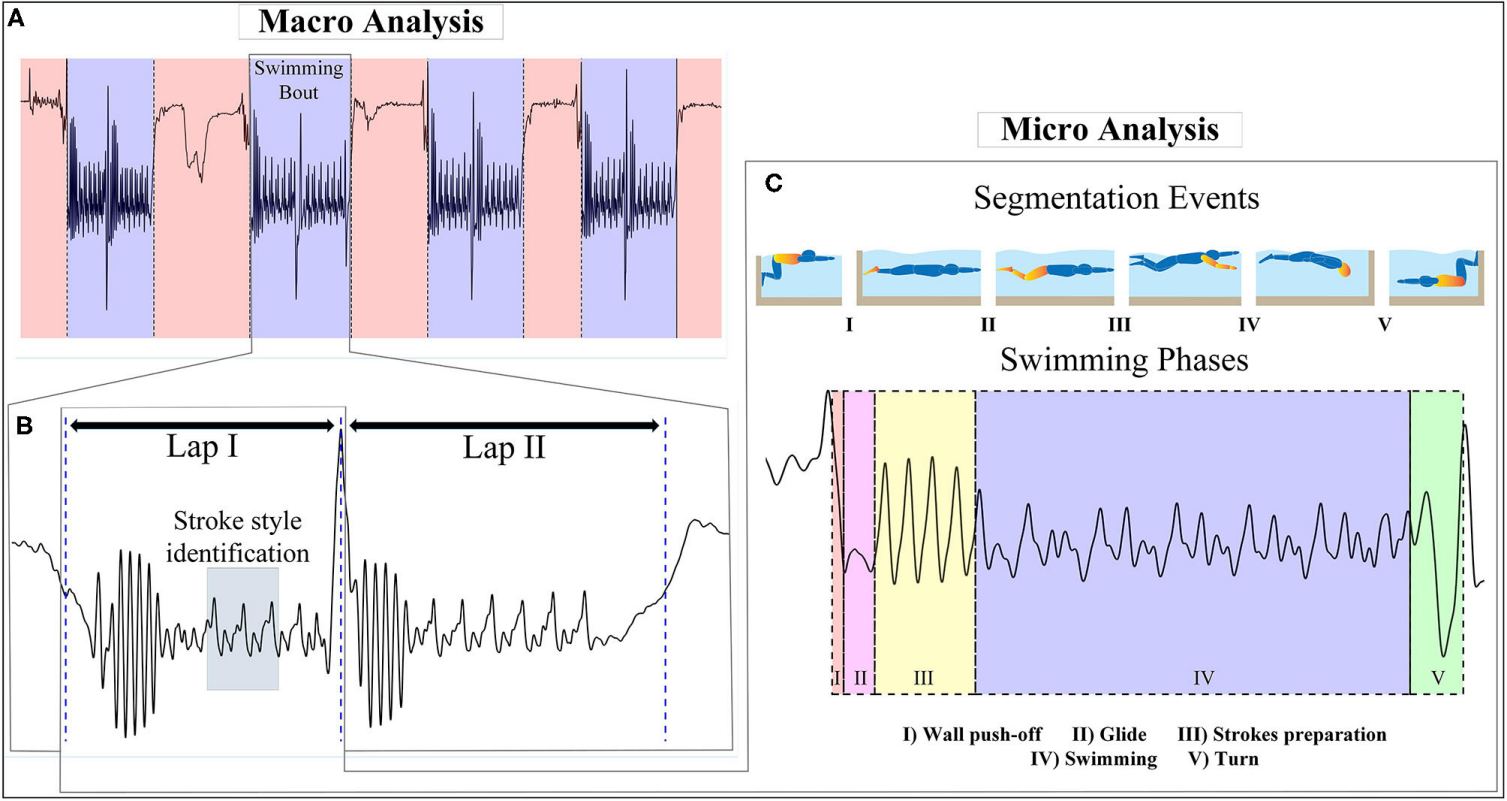
Analysis approach and segmentation events considered in this study (sacrum acceleration signal during frontcrawl is used as an example). The approach steps are: **(A)** swimming bouts detection in a training session, **(B)** laps separation and swimming technique identification using a short period of upper limbs cycles, **(C)** segmentation of the lap into five swimming phases of wall push-off, glide, strokes preparation, swimming, and turn using the segmentation events.

The training session can be conceptualized at a macro level estimating the volume of training, i.e., number and duration of swimming bouts and laps with a specific swimming technique, and at a micro level including different phases of each lap as well as spatiotemporal features of swimming within each phase (number, duration, or distance per stroke). Here, macro analysis consists of swimming bouts detection, laps detection, and swimming technique identification, while micro analysis is limited to phase detection within each lap (Figure 4) and more detailed parameters in each phase is not included in this study. As these phases follow each other sequentially, we focused on finding the beginning of each phase for lap segmentation. The start and end of each phase triggers specific change in the profile of acceleration and angular velocity of body segment and requires specific rules for its detection, the details of which are discussed in Supplementary Tables 1, 2. These rules are based on common processing functions described in the following section.

### Common Processing Functions

Despite the differences between the movement patterns of body segments, there are common function that are used frequently in macro-micro analysis algorithms. These functions are explained in Table 2 and applied on acceleration (*Acc*_*x*_, *Acc*_*y*_, *Acc*_*z*_) and angular velocity (*Gyr*_*x*_, *Gyr*_*y*_, *Gyr*_*z*_) or their norms (*|Acc|* and *|Gyr|*) expressed in the bone anatomical frame after noise removal with low-pass filtering (second order Butterworth filter, *f*_*c*_ = 10 Hz). These methods are thresholding (Cronin and Rumpf, 2014), extremum detection (Chardonnens et al., 2012), sharp change detection (Dadashi et al., 2013a), principle component Analysis (Jollife and Cadima, 2016), frequency analysis (Aung et al., 2013), empirical mode decomposition and Hilbert-Huang transform (Ge et al., 2018).

**Table 2:** Common processing methods used for macro-micro analysis.

**Method**	**Description**	**Example**
Thresholding (TH)	When the signal goes higher or lower than a threshold (TH) due to an event, thresholding can detect it.	Acceleration amplitude change for swimming bouts detection on wrist
Extremum detection [A, t] = EXT(s, TH)	Local increase or decrease of the signal (s) generates peaks or troughs comparable with a threshold (TH). Extremum detection finds the magnitude (A) and time (t) of extremums	Peak on sacrum forward acceleration at the beginning of wall push-off phase
Sharp change detection t = SC(s, TH)	The occurrence time (t) of some events are abrupt, easier to detect on the derivative of the signal (s) by comparing it with a threshold (TH)	Swimming bout start and end detection with sacrum
PCA analysis [PC_1_, PC_2_, PC_3_] = PCA(v)	Finding the principle components (PC_1_, PC_2_, PC_3_) of a vector (v) is useful for decreasing dimensionality to identify the type of movement	Swimming technique identification with head
Frequency analysis FFT(s)	The single-sided power density spectrum of the signal (s) and its analysis reveals the behavior of the signal in frequency domain	Differentiating between breaststroke and butterfly techniques with sacrum
Empirical mode decomposition IMF = EMD(s)	It decomposes the signal (s) into its intrinsic modes to facilitate the change detection in time domain	Detecting the beginning of upper limbs cycles on sacrum
Hilbert-Huang transform inse = HHT(s)	It extracts many features of a signal (s) such as instantaneous energy, which is useful to find the change start	Detecting the beginning of upper limbs cycles on head

For macro-micro analysis algorithms, a mixture of these methods are used for all sensor locations. As most of the motions are symmetric, always the sensor on the right wrist and shank are used in algorithms unless mentioned otherwise. The details of macro and micro algorithms are explained in Supplementary Tables 1, 2.

### Macro Analysis Algorithms

#### Swimming Bouts Detection

Each swimming bout starts and ends with an abrupt change in swimmer's body posture between upright and supine or prone postures. This change is observed either after (for swimming bout start) or before (for swimming bout end) a rest period. The detection method on all sensor locations except right wrist is to use *SC*(*Acc*_*y*_, *TH*_*B*_), where *TH*_*B*_ = ± 0.3 × *EXT*(low pass filtered $\dot{Acc_{y}}$), where $\dot{Acc_{y}}$ denotes the derivative of *Acc*_*y*_. Negative and positive threshold is used for detecting troughs (corresponding to approximate start) and peaks (corresponding to approximate end), respectively.

(1) $$\begin{array}{l} t=SC\left(Acc_{y},0.3\;\times EXT\left(\text{Low pass filtered }\dot{Acc_{y}}\right)\right) \end{array}$$

(2) $$\begin{array}{l} \text{Approximate start}=\text{t }\left(\text{negative peaks on }{\dot{Acc}}_{y}\right) \end{array}$$

(3) $$\begin{array}{l} \text{Approximate end}=\text{t }\left(\text{positive peaks on }{\dot{Acc}}_{y}\right) \end{array}$$

For the right wrist sensor, while it has a clear cyclic pattern during swimming phase in all swimming techniques, its motion is erratic before upper limbs actions. Despite the inter-individual variability in swimmer's wrist motion during swimming phase (Martens et al., 2016), the swimming bout was detected as the period where the envelope of |*Acc*| is higher than an empirical threshold (*TH*_*BW*_ = 1.6 g). This period starts with the upper limbs cycles in the first lap, until the end of the swimming bout.

#### Lap Detection

In our measurement protocol, each swimming bout consisted of two laps, separated by a turn. Therefore, lap detection requires finding the approximate turn. The detection algorithm for sacrum, head, and right shank finds the highest peak during the swimming bout on *Acc*_*x*_ and |*Acc*_*y,z*_|. For right shank the peak is detected using a threshold with the function *EXT*(*Acc*_*z*_ *or Gyr*_*z*_ (the one happens earlier), *TH*_*LS*_ = highest peak in a 2-s period during swimming phase). As wrist's angular velocity amplitude decreases during turns (compared to swimming phase), the algorithm detects a decrease of |*Gyr*| where low pass filtered |*Gyr*| is less than the threshold (*TH*_*LW*_ = 200 °/s).

#### Swimming Technique Identification

For head and sacrum, a two-upper-limbs-cycle period was chosen. The *PCA(Gyr*_*x,y,z*_) to separate swimming techniques with dominant trunk pitching motion (breaststroke/butterfly) from the techniques with trunk rolling motion (front crawl/backstroke), gravity effect (positive vs. negative sign of *Acc*_*x*_ average to distinguish backstroke) and threshold-based Fast Fourier Transform (*FFT*) of *Acc*_*x*_ for sacrum and |*Acc*_*x,y*_| for head (to distinguish between butterfly and breaststroke) were used for swimming technique identification (*TH*_*StyleHE*_ = 0.2 g, *TH*_*StyleSA*_ = 0.16 g). Equation (4) explains the use of *FFT* analysis for technique identification on sacrum and head.

(4) $$\begin{array}{l} EXT\left(\text{power density spectrum magnitude},TH_{StyleHE}\text{ or }TH_{StyleSA}\right)\\ \;\to \text{Butterfly technique} \end{array}$$

A period including five lower limbs actions is chosen during swimming phase for swimming technique identification with right shank, which was not a limit, as all of the swimmers did more than five lower limbs actions in every lap. Gravity effect (same as head and sacrum to distinguish backstroke) and *PCA* analysis of angular velocity vector are used for swimming technique identification on right shank. For right wrist, the *PCA* of acceleration separates backstroke from other techniques and the mean and variation of |*Acc*| are compared with two thresholds (*TH*_*StyleWmean*_ = 1.7 g, *TH*_*StyleWvar*_ = 0.01 g) to identify butterfly and front crawl, respectively.

### Micro Analysis Algorithms

The results achieved from macro analysis (approximate start, approximate end, approximate turn, and swimming technique) were used for further detailed lap components detection. These approximate events are enough to find the exact locations of the events for phase detection in micro level.

#### Beginning of Wall Push-Off (**Push**_**B**_)

Wall push-off accompanies a forward acceleration increase close to approximate start. For sacrum and head during backstroke, the detection is done with *EXT(Acc*_*y*_*)* for both sensor locations, while for other techniques with sacrum, concavity change of *Acc*_*y*_ is used to find a negative trough, close to *Push*_*B*_. For head during other swimming techniques, *EXT(*|*Acc*|*)* estimates the answer. Right wrist has a downward motion during wall push-off causing a negative trough on *Acc*_*y*_ and right shank represents a peak on |*Gyr*| close to *Push*_*B*_.

#### Beginning of Glide (**Glid**_**B**_)

As the glide phase starts, the whole body glides in water with no propulsion. The first trough after *Push*_*B*_ detected by *EXT*(-*Acc*_*y*_) for sacrum and head or the first peak found by *EXT*(|*Gyr*|) for right shank is considered as *Glid*_*B*_. On the right wrist, *Acc*_*y*_ gets close to zero and shows a peak right after beginning of wall push-off, which is *Glid*_*B*_.

#### Beginning of Strokes Preparation (**StPr**_**B**_)

Strokes preparation phase includes underwater lower limbs actions (except for breaststroke, which includes one lower limb action and one upper limb cycle). Detection method for sacrum, head, and right wrist is threshold-based and the idea is using thresholds on peak magnitude, peak prominence or signal variation depending on sensor location (for sacrum; *EXT*(|*Acc*_*x*_|, *TH*_*SPSA*_ = g, *TH*_*SPSAvar*_ = 0.06 g), for head; *EXT*(|*Acc*_*y*_|, *TH*_*SPHE*_ = −0.5 g, *TH*_*SPHEprom*_ = 0.1 g), for right wrist; *EXT*(|*Acc*|, *TH*_*SPRW*_ = −0.9 g). On right shank, the first positive peak of *Acc*_*y*_ is *StPr*_*B*_ for backstroke, while for other swimming techniques, the peak is detected with *EXT*(|*Acc*_*x*_|, *TH*_*SPRS*_ = 1.3 g) and then the next sample on |*Acc*| passing from g is *StPr*_*B*_.

#### Beginning of Swimming (**Swim**_**B**_)

In swimming phase, swimmer's body starts the rolling (for front crawl and backstroke techniques) or pitching motion (for breaststroke and butterfly techniques). On sacrum, the detection for front crawl and backstroke is done using *EXT*(|*Gyr*_*y*_|, *TH*_*SSA*−*FCBaS*_ =200 °/s). For breaststroke and butterfly, the second intrinsic mode of low pass filtered *Gyr*_*z*_ and *Acc*_*y*_ were obtained. For breaststroke, instantaneous energy of *Gyr*_*z*_ increases more than *TH*_*SSA*−*BrS*_ =550 °/s^2^ at *Swim*_*B*_. For butterfly, *EXT* (second intrinsic mode of *Acc*_*y*_, *TH*_*SSA*−*BF*_ = 0.1 g) detects a peak close to *Swim*_*B*_. On head, instantaneous energy of *Gyr*_*y*_ (for front crawl) and *Gyr*_*z*_ (for breaststroke, butterfly, and backstroke) are used. The decrease (for backstroke) or increase (for front crawl, breaststroke, and butterfly) of instantaneous energy is taken as the criterion for *Swim*_*B*_ detection by thresholds *TH*_*SHE*−*FC*_ = 5,000 °/s^2^, *TH*_*SHE*−*BFBrS*_ = 12,000 °/s^2^ and *TH*_*SHE*−*BaS*_ = 1,000 °/s^2^.

For wrists during front crawl and backstroke, both wrists are used to find *Swim*_*B*_ because upper limbs cycles can start on either one. The detection method is to find the trough before the first peak on right and left wrists. It is performed over *Acc*_*y*_ for front crawl and butterfly and over *Acc*_*x*_ for backstroke. The same is done over *Gyr*_*y*_ for breaststroke to find an approximation of *Swim*_*B*_. On right shank, the lower limbs action pattern changes after *Swim*_*B*_, which is noticeable on the second intrinsic mode of *Acc*_*x*_ (for front crawl, butterfly, and backstroke) or *Acc*_*y*_ (for breaststroke). The trough before the first peak found with *EXT*(second intrinsic mode of *Acc*_*x*_ or *Acc*_*y*_, *TH*_*T*−*RS*_ = 1.7 g) is considered as *Swim*_*B*_.

#### Beginning of Turn (**Turn**_**B**_)

Regardless of the turn type (simple or tumble turn), the algorithms use approximate turn to find the beginning of turn. During backstroke, approximate turn fits greatly as *Turn*_*B*_. For the rest of the techniques, *Turn*_*B*_ on sacrum is the first trough before the large peak on *Acc*_*x*_ close to approximate turn. On head, *EXT*(|*Acc*|) and *EXT*(*Gyr*_*x*_) were used shortly before approximate turn for tumble turn and simple turn, respectively, to find *Turn*_*B*_. On right wrist, *EXT*(low pass filtered *Acc*_*y*_) and *EXT*(*Acc*_*y*_) are used for tumble turn and simple turn, respectively, to find *Turn*_*B*_. Right shank motion also shows a peak detectable, respectively, by *EXT*(*Gyr*_*z*_) and *EXT*(*Acc*_*z*_) for tumble turn and simple turn.

### Validation and Error Analysis

For validating the temporal macro and micro events described above, cameras were used as ground truth. To validate the macro events the camera over water was used as the main reference, while the detection of swimming phases start during micro analysis was done by underwater cameras by one observer. For validation of swimming bouts and laps detection, the accuracy, sensitivity, and precision are defined based on the number of true or false detections (Equations 5–7). Accuracy shows how much the algorithms work correctly and the results match the true values. Precision represents how much the algorithm results are correct when it claims the detection of an event (if it is truly happened or not), and sensitivity displays how much the algorithm is sensitive to occurrence of an event (if it is correctly detected or not)

(5) $$\begin{array}{l} \text{Accuracy}=\frac{\sum \text{True positive}+\sum \text{True negative}}{\sum \text{Total}} \end{array}$$

(6) $$\begin{array}{l} \text{Sensitivity}=\frac{\sum \text{True positive}}{\sum \text{True positive}+\;\sum \text{False negative}} \end{array}$$

(7) $$\begin{array}{l} \text{Precision}=\frac{\sum \text{True positive}}{\sum \text{True positive}+\;\sum \text{False positive}} \end{array}$$

For example, the results are checked if the beginning and end of a swimming bout or turns are correct (true positive), missed (false negative), or mixed with other motions (false positive). Total parameter includes all the cases (e.g., the number of all the turns) and true negative is zero for our algorithms, as the purpose is to detect the happening of the event. The same logic holds true for swimming technique identification, if the technique is correctly identified or mixed with another technique.

Synched with the IMUs, the cameras were used to mark the frames when each phase started and finished. The detected event using IMUs was then compared to the corresponding frame on the cameras and the mean and standard deviation of the errors were calculated. This method is used for validation in swimming for comparing IMUs and cameras in similar studies (Dadashi et al., 2013b). To assess the reliability of the validation process, two observers detected the events on cameras and compared with each other using Bland-Altman plots for the beginning of each phase. For each event, mean and standard deviation of the difference between the event observed on camera and IMU were calculated.

For phase duration (denoted by Δ of the phase name, e.g., Δ*Push* for wall push-off phase duration) confined with its starting and ending event, the absolute and relative error of phase duration are calculated. This error is the difference of estimated duration and the true duration (obtained from validation system). The relative phase duration error is then calculated by dividing it to the true phase duration. Equations (9) and (10) are examples for *Push* phase duration error and relative error. Δ*Push* denotes the duration of *Push* phase, Δ*Push*_*IMU*_ signifies the duration of *Push* phase estimated by IMUs and Δ*Push*_*True*_ is the duration of *Push* phase estimated by cameras. Then mean and standard deviation of phase duration error and phase duration relative error were calculated.

(8) $$\begin{array}{l} \Delta Push\;=Glid_{B}-Push_{B} \end{array}$$

(9) $$\begin{array}{l} \text{Phase duration error}=\;\Delta Push_{IMU}-\;\Delta Push_{True} \end{array}$$

(10) $$\begin{array}{l} \text{Phase duration realive error},\;\%=\frac{\Delta Push_{IMU}-\;\Delta Push_{True}}{\Delta Push_{True}} \end{array}$$

Three swimmers were chosen randomly from the dataset (one female and two males, making 20% of the dataset) who were trained with different coaches and tested in different pools. These swimmers were from the same technique level as others and trained regularly as planned by coaches. To make the algorithms more generalizable, they were developed using the data from these three swimmers and then tested over the other 14 swimmers to include as much diversity as possible in the algorithms.

All of the algorithms that use threshold have been analyzed in terms of their results sensitivity to the change of threshold values. The results are the accuracy and precision for macro analysis algorithms and the error mean and standard deviation for micro analysis algorithms. The table including the exact values is presented in Supplementary Table 3. Each threshold is changed at least 10% in both directions and the corresponding effect on algorithm results have been explored.

## Results

In order to generate the results, the data of all laps are used for swimming technique identification and the phases are investigated from the beginning of each swimming bout up to the end of the turn to have all the phases completely.

### Macro Analysis Results

**Figure 5:**
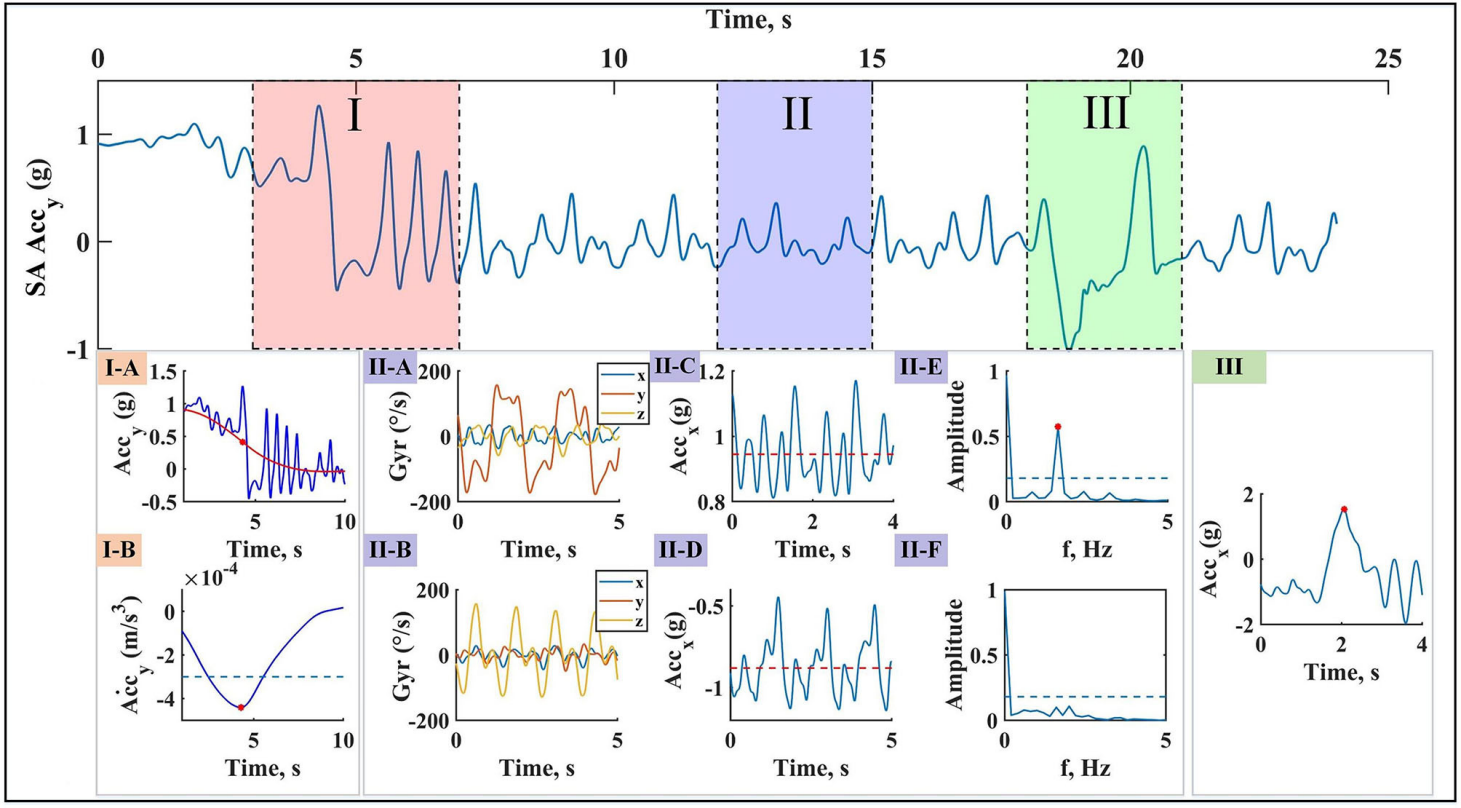
Example of macro analysis with sacrum **Acc**_**y**_ data. **(I)** Swimming bouts detection: swimming bout start causes a change in filtered **Acc**_**y**_ amplitude level (I-A), detected using its derivative (I-B) and the rule is the same for swimming bout end. **(II)** Swimming technique identification: a short period of upper limbs cycles is selected for swimming technique identification. Principal component of angular velocity (II-A for front crawl or backstroke, II-B for breaststroke or butterfly), gravity effect on **Acc**_**x**_ (II-C for front crawl, breaststroke, or butterfly, II-D for backstroke), and FFT of the data (II-E for butterfly, II-F for breaststroke) are mainly the tools used for this purpose. **(III)** Lap detection: at the end of each lap, turning accompnies with a peak on **Acc**_**x**_.

Figure 5 shows a typical example of macro analysis using sacrum sensor. As described in section Macro Analysis Algorithms, posture changes at the beginning and end of swimming bout were detected by the filtered $\dot{Acc_{y}}$ (Figures 5I-A,B). The approximate turn within each swimming bout are detected for separating laps (Figure 5III-A). Swimming techniques were identified based on *PCA*(*Gyr*_*x,y,z*_), gravity effect of *Acc*_*x*_ and dominant frequency during a period of swimming phase (Figures 5II-A–F). It is worth mentioning that the frequency resolution of fast Fourier transform analysis was at least 0.35 Hz considering all swimmers and swimming techniques, small enough to capture the dominant frequency.

**Figure 6:**
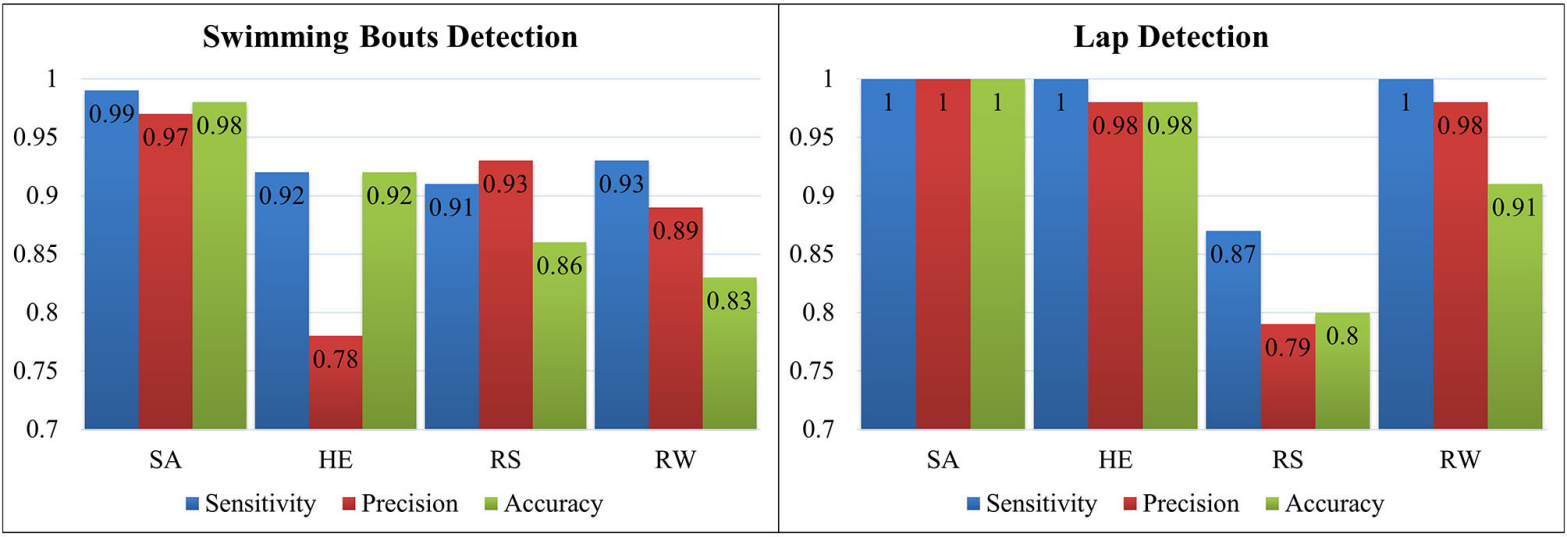
Sensitivity, precision and accuracy achieved for swimming bouts and laps detection on all sensor locations (SA, HE, RS, and RW).

According to Figure 6, sacrum shows the most promising results in terms of both accuracy and precision for swimming bouts and lap detection. After lap detection, the swimming technique is identified with each sensor separately (Table 3). Sacrum represents the best results for all swimming techniques. It is possible to identify all front crawl and backstroke laps correctly and differentiate between breaststroke and butterfly with precision and accuracy higher than 0.97.

**Table 3:** Accuracy and precision for swimming technique identification of four swimming techniques over different sensor locations (SA, HE, RS, and RW).

**Sensor location**	**SA**				**HE**			
**Swimming technique**	**Front crawl**	**Breaststroke**	**Butterfly**	**Backstroke**	**Front crawl**	**Breaststroke**	**Butterfly**	**Backstroke**
Precision	1.00	0.98	0.97	1.00	1.00	0.86	0.83	1.00
Accuracy	1.00	0.97	0.98	1.00	0.99	0.82	0.86	0.97
**Sensor location**	**RS**				**RW**			
**Swimming technique**	**Front crawl**	**Breaststroke**	**Butterfly**	**Backstroke**	**Front crawl**	**Breaststroke**	**Butterfly**	**Backstroke**
Precision	0.80	0.86	0.93	1.00	0.77	0.76	0.91	0.79
Accuracy	0.91	0.81	0.82	1.00	0.81	0.73	0.90	0.86

### Micro Analysis Results

**Figure 7:**
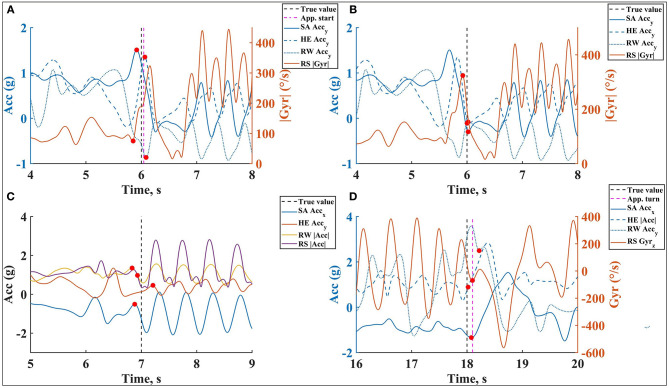
An example of the swimming phases beginning event detection, **(A)** **Push**_**B**_, **(B)** **Glid**_**B**_, **(C)** **StPr**_**B**_, **(D)** **Turn**_**B**_, on all sensor locations during front crawl. The estimated values are represented on the corresponding signal with red dots and the true value is shown as a vertical dashed line.

**Figure 8:**
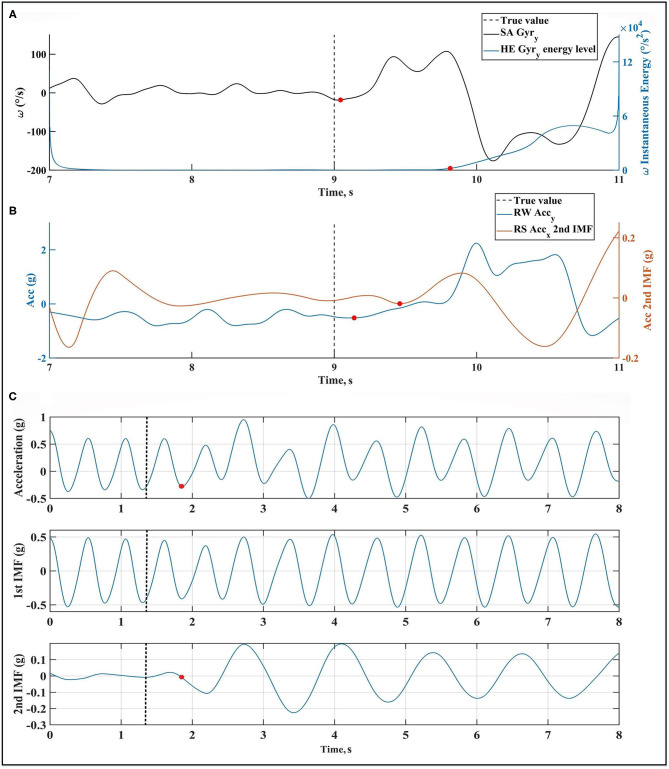
An example of **Swim**_**B**_ event detection on all sensor locations during butterfly. **(A)** **Swim**_**B**_ detection on SA and HE during front crawl, **(B)** **Swim**_**B**_ detection on RW and RS during front crawl, **(C)** an example of the process of using EMD technique for **Swim**_**B**_ detection during butterfly technique. It is shown that the second intrinsic mode function (IMF) separates the motion after **Swim**_**B**_. The estimated values are represented on the corresponding signal with red dots.

Figures 7, 8 show one example of detecting the beginning of these events on corresponding locations and signals. The examples show the estimated values on different locations (red dots) are close to each other and to the true value (the black dashed line), such as beginning of wall push-off, whereas estimations are more diverse for some other events, such as swimming start. The main challenge is whether or not the phase starts at the same time on all sensor locations and which limb is used to define the beginning of the phase. The mean and standard deviation of error for the beginning of each phase on all sensor locations are displayed in Table 4.

**Table 4:** Phases starting event detection error in ms by comparing IMU and camera results on all sensor locations (SA, HE, RS, and RW).

**Phase event**	**Push_B_**	**Glid_B_**	**StPr_B_**	**Swim_B_**	**Turn_B_**	**Push_B_**
SA	−20 ± 89	4 ± 100	−32 ± 107	136 ± 226	23 ± 97	−1 ± 65
HE	−35 ± 76	−35 ± 58	87 ± 214	58 ± 563	53 ± 195	−1 ± 70
RS	−118 ± 77	76 ± 77	0 ± 88	342 ± 473	−47 ± 390	−64 ± 89
RW	40 ± 71	49 ± 51	−151 ± 124	−42 ± 72^*^	118 ± 151	44 ± 82

*The events are **Push**_**B**_, **Glid**_**B**_, **StPr**_**B**_, **Swim**_**B**_ and **Turn**_**B**_ and the next **Push**_**B**_, which completes the lap segmentation*.

* *Obtained using both right and left wrists*.

The accuracy of detecting each event changes with the sensor location and type of event. Based on the results, right shank has the highest error mean at the beginning of the lap (for beginning of wall push-off and beginning of glide) where the motion is the same for all swimming techniques. However, right shank provides an estimation with lowest error mean and standard deviation for beginning of strokes preparation, while it is detected with negative (on right wrist and sacrum) or positive (on head) error mean on other sensor locations. Beginning of swimming seems to be the most challenging event since the mean and standard deviation of error is high on all locations other than right wrist, where the swimming phase starts. Although beginning of turn results depend on turn type, sacrum and head both estimate it with low error mean and standard deviation.

Although the results depend on swimming technique, they match with the detected events displayed in Figures 7, 8. The mean and standard deviation of absolute and relative error for each phase duration (Δ*Push*, Δ*Glid*, Δ*StPr*, Δ*Swim*, Δ*Turn*) over all sensor locations are displayed in Table 5. Depending on the duration of each phase, error percentage vary based on the sensor location. For short phases (such as wall push-off), the relative error is higher than long phases, since even a small error will cause a high relative error in phase duration estimation. To provide a comparison between four swimming techniques in terms of micro analysis results, the range of micro analysis error is reported in Table 6. The table represents the range of both error mean (mean range) and standard deviation (standard deviation range) for four techniques.

**Table 5:** Estimated phase duration (with IMU), its phase duration error and phase duration relative error compared to the true phase duration (with camera) for each sensor location (SA, HE, RS, and RW).

**Phase duration**		***ΔPush***	***ΔGlid***	***ΔStPr***	***ΔSwim***	***ΔTurn***
True values (validation data)		218 ± 29	880 ± 476	2,673 ± 1,268	12,423 ± 1,905	1,223 ± 166
SA	Estimated (mean ± SD)	242 ± 37	991 ± 560	2,732 ± 1,439	12,241 ± 1,754	1,180 ± 170
	Error (mean ± SD)	22 ± 51	10 ± 218	152 ± 300	−100 ± 286	−26 ± 69
	Relative error (mean ± SD)	12 ± 24	−1 ± 24	4 ± 12	−0.8 ± 2	−2 ± 5
HE	Estimated (mean ± SD)	211 ± 52	936 ± 442	2,424 ± 1,185	12,263 ± 2,700	1,069 ± 355
	Error (mean ± SD)	−7 ± 53	121 ± 218	−27 ± 1,124	−14 ± 1,255	−149 ± 334
	Relative error (mean ± SD)	−2 ± 25	8 ± 27	−1 ± 42	0.6 ± 9	−11 ± 26
RS	Estimated (mean ± SD)	415 ± 70	815 ± 470	3,093 ± 1,127	10,812 ± 2,873	1,198 ± 390
	Error (mean ± SD)	199 ± 80	−82 ± 113	479 ± 737	−767 ± 1,096	−2 ± 393
	Relative error (mean ± SD)	96 ± 46	−7 ± 15	21 ± 35	−6 ± 8	−3 ± 29
RW	Estimated (mean ± SD)	204 ± 43	775 ± 460	2,730 ± 1,234^*^	12,358 ± 1,732^*^	1,082 ± 225
	Error (mean ± SD)	1 ± 46	−188 ± 184	118 ± 147^*^	154 ± 190^*^	−122 ± 170
	Relative error (mean ± SD)	2 ± 22	−21 ± 19	5 ± 6^*^	1 ± 1^*^	−10 ± 17

*All value are expressed in ms except for relative error expressed in percent*.

* *Obtained using both right and left wrists*.

**Table 6:** The range of error mean (Mean range) and standard deviation (SD range) during micro analysis using four sensor locations (SA, HE, RW, and RS) in four swimming techniques.

	**SA**		**HE**		**RW**		**RS**	
	**Mean range**	**SD range**	**Mean range**	**SD range**	**Mean range**	**SD range**	**Mean range**	**SD range**
Front crawl	78	123	421	346	234	106	427	322
Breaststroke	314	63	516	306	427	88	358	595
Butterfly	287	109	152	384	411	37	569	390
Backstroke	154	186	413	503	455	180	723	306

*The values are in millisecond*.

**Figure 9:**
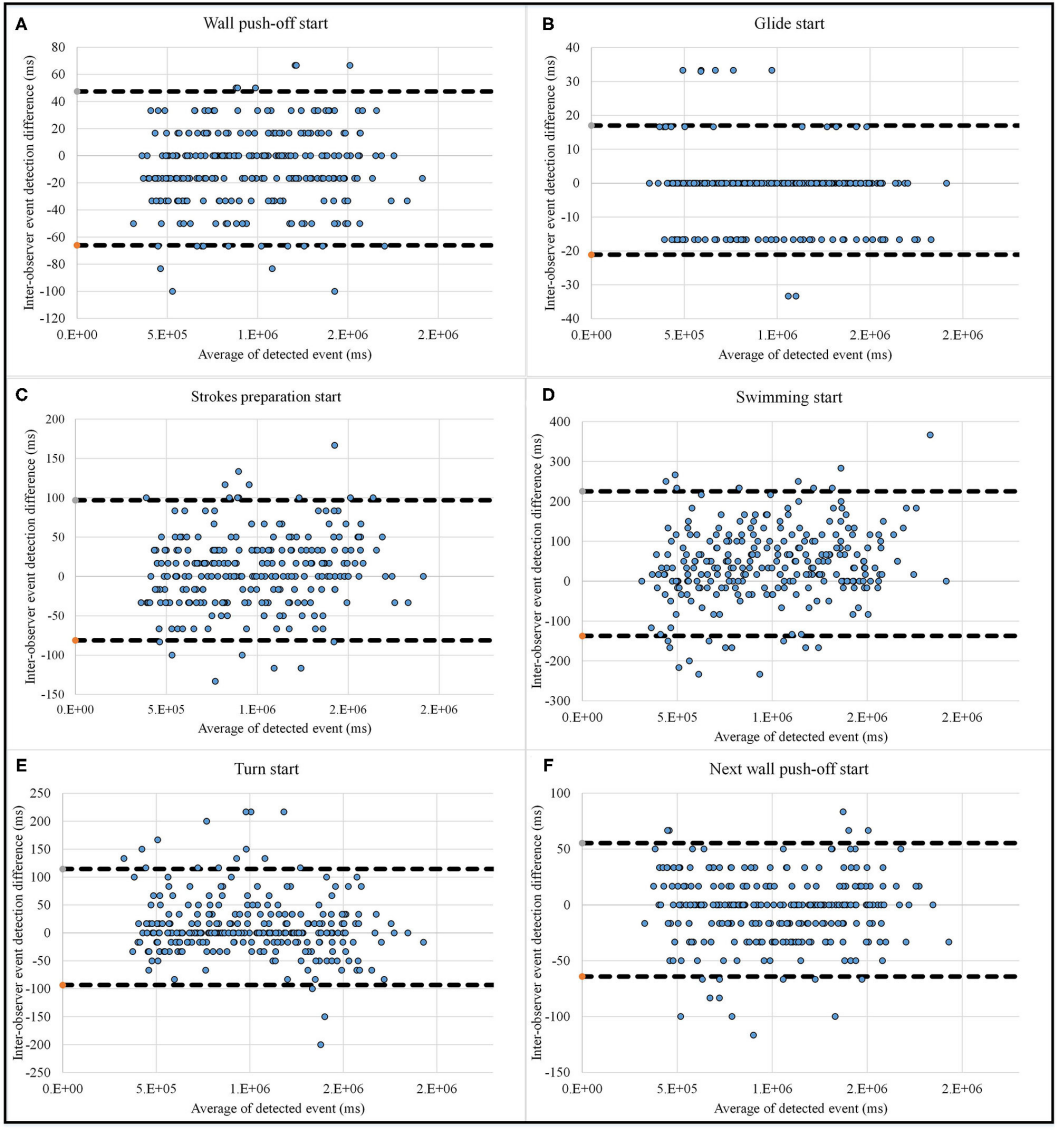
Bland-Altman plot for inter-observer agreement for micro analysis event detection, including wall push-off start **(A)**, glide start **(B)**, strokes preparation start **(C)**, swimming start **(D)**, turn start **(E)** and next wall push-off start **(F)**, which completes the lap.

In order to check the reliability of the validation method, the true frames on cameras are detected with a second observer and compared with the first observer using Bland-Altman plots. Figure 9 show the agreement between two observers with a 95% limit of agreement (*LoA*). The plots show that the limit of agreement is higher for swimming start (225 ms), turn start (115 ms), and strokes preparation start (100 ms), while it is lower than 65 ms for other phases.

All of the thresholds have been modified at least 10% depending on their values, while the results changed <5% for all of them except for *TH*_*SPHE*_ and *TH*_*SPHEprom*_, which changed the estimated beginning of strokes preparation with head result more than 10%, meaning that they should be chosen more carefully.

## Discussion

In this study, a novel swimming analysis method with a macro-micro approach was proposed, which applies the same unified analysis for all main techniques. Our hypothesis was that adequate IMU-based algorithms are capable of studying a training session both in macro and micro levels, confirmed by the achieved results. These results were presented in terms of accuracy and precision to find the most suitable sensor location for this approach. In order to have a higher sample size, we did not separate male and female swimmers. Although the algorithms used only right shank or right wrist, the same results are likely to be achieved with left shank or left wrist because of motion similarity. The range of swimming velocity during the tests for front crawl, breaststroke, butterfly, and backstroke are [1.5–1.9], [1.0–1.4], [1.3–1.7], and [1.3–1.7] m/s, respectively. As a result, the algorithms and discussion are valid for these paces.

### Macro Analysis

Starting with macro analysis from swimming bout detection, sacrum has the best results among all locations (sensitivity = 0.99, precision = 0.97, accuracy = 0.98). Located closer to body center of mass, sacrum and head motions are more distinguishable among the sensor locations for macro analysis and more robust for swimming bouts detection based on our analysis method. Our algorithm cannot distinguish between head motion during simple turns and the swimming bout start in some cases, which decreases the algorithm precision (precision = 0.78).

Sacrum and head reached the best results for lap detection. Right shank achieves worse lap detection results (sensitivity = 0.87, precision = 0.89, accuracy = 0.80) than sacrum or head because it is less affected by the sudden change of acceleration pattern due to turn rapid dynamics. Lap detection with right wrist works during the swimming bouts starting from first lap swimming phase, which is a downside for this location. As a result, lap detection algorithm with right wrist worked on a shorter period and reached better results (sensitivity = 1.00, precision = 0.98, accuracy = 0.91) than right shank. Previous studies only focused on lap detection on sacrum (Le Sage et al., 2011) and head (Jensen et al., 2013) and reached lower accuracies than ours.

The results of swimming technique identification show that sacrum is the most reliable sensor location which identifies front crawl and backstroke correctly and has an accuracy and precision higher than 0.95 for breaststroke and butterfly. Right wrist motion is the most variant among swimmers and resulted in the worst results. It is well-established that hand movement pattern shows significant variances owing to various factors including individual anthropometric and technique differences or skill level (Seifert et al., 2011b). Moreover, inter-cyclic variation is another important factor (Barbosa et al., 2005; Figueiredo et al., 2012) which might cause error in technique identification, which was not examined in this study. Our method, however, presents a higher accuracy in comparison to reported result in the literature based on sacrum sensor (Davey et al., 2008; Omae et al., 2017). Some studies use a network of IMUs (Wang et al., 2016) or a smartphone (Pan et al., 2016) for swimming technique identification while we focused on each sensor location separately.

### Micro Analysis

Running a Wilcoxon rank sum test over the segmentation error of male and female swimmers showed that there is no significant difference (*p* > 0.05) between them and the results can be mixed. Lap segmentation results are presented in Table 4. Starting from *Push*_*B*_, the algorithms developed for sacrum and head achieved lower error mean and standard deviation. Since *Push*_*B*_ is defined as the beginning of trunk forward motion, these two locations are more suitable to capture it. Error mean is negative and higher on right shank both for first (−118 ms) and second (−64 ms) wall push-off (the *Push*_*B*_ after turn). This is because during wall push-off phase, swimmer starts extending shanks for push-off during body posture change from vertical to horizontal before sacrum forward motion.

*Glid*_*B*_ is detected with the lowest and highest error mean on sacrum (4 ms) and right shank (76 ms), respectively. As sacrum, right wrist and head are located superior to right shank, the transition between wall push-off to glide phase happens more abruptly in these locations, whereas the right shank angular velocity change is smoother (the peak of |*Gyr*| is difficult to observe in some cases) on glide start.

*StPr*_*B*_ is detected earlier on right wrist (−151 ms) and the error standard deviation is high for head (214 ms) and right wrist (124 ms), while right shank shows the lowest error mean and standard deviation. Strokes preparation phase accompanies with generating a wave in the whole body after glide phase. This wave starts from right shank by the first lower limb action but for many swimmers, wrist motion happens earlier for reaction force generation during lower limb actions, resulting in high negative error for right wrist. As the wave starts in lower limb or upper limb, the error standard deviation for the sensor attached to the upper limb increases (right wrist and head). Located in the middle of this wave, sacrum captures the motion with a moderate error mean and standard deviation (−32 ± 107 ms).

Since *Swim*_*B*_ is defined as upper limbs cycle start on hand, wrists obtain the best result (−42 ± 72 ms). During front crawl or backstroke, sacrum is delayed (136 ms), sometimes two or three upper limbs cycles, in receiving the rolling motion during swimming phase, which is used for *Swim*_*B*_ detection. Right shank is also delayed (342 ms) mostly during butterfly or breaststroke techniques since lower limb action starts always after the upper limbs cycle on hand. High standard deviation for swimming start detection on sacrum (226 ms), head (563 ms), and right shank (473 ms) are the result of high variation between swimmers and motion transfer delay to these sensor locations. For example, the lower limbs action might start after or before upper limbs cycle during front crawl and backstroke as it is not dependent on upper limbs.

Although the detection of *Turn*_*B*_ relies mainly on turn type (simple or tumble turn), sacrum is the best location for it (23 ± 97 ms), as the turn motion reaches sacrum right after it starts on head (tumble turn) or wrist (simple turn). Right wrist has a late response during tumble turn, which causes high positive error mean (118 ms) since the swimmer tries to keep wrists backward and right wrist does not necessarily follow the turn quick motion. The wall reaching speed also affects the standard deviation of *Turn*_*B*_ detection with right shank (390 ms) and head (195 ms). The swimmer should estimate the wall distance at the right time before turn and adapt their speed. When the swimmer touches the wall with low or high speed in simple turn, the algorithms detect *Turn*_*B*_ on head and right shank earlier or later than the true value.

To understand better the event detection error, the estimated phase duration and its absolute and relative error compared to the true value are shown in Table 5. Detecting the phase duration for short phases accompanies with higher relative error. For example, this value for Δ*Push* detection on sacrum is 12 ± 24%, while the same value for Δ*Swim* on sacrum is −0.8 ± 2%. Hence, the detection of long phases duration such as swimming phase is more reliable than short phases. The absolute value of each phase duration error is affected by both phase start and end detection error. As shown in Table 5, right wrist has the highest amount of error for Δ*Swim* estimation, while it was the best location for *Swim*_*B*_ detection, the reason of which is its poor performance for *Turn*_*B*_ detection. Although the short phases duration estimation has higher relative error, the parameters within these phases are possible to extract. Interesting parameters, such as maximum push-off velocity (Stamm et al., 2013a) during wall push-off lies between wall push-off start and end. The superiority of sacrum for micro analysis over other sensor locations is pointed out by the results displayed in Table 6.

The smallest range of error mean (78 ms for front crawl, 314 ms for breaststroke, 287 ms for butterfly, and 154 ms for backstroke) and standard deviation (123 ms for front crawl, 63 ms for breaststroke, 109 ms for butterfly, and 186 ms for backstroke) for all swimming techniques are achieved with sacrum. In conclusion, this location is the best for micro analysis in all swimming techniques. Since sacrum also worked better in macro level, this is the best candidate for a single sensor analysis system. In macro scale, sacrum data can provide reliable results, and in micro level, it captures the events starting from upper limbs and lower limbs with less delay than other sensor locations, as it is located in the middle of the body. As shown by Bland-Altman plots (Figure 9), the inter-observer limit of agreement is 225, 115, and 100 ms for beginning of swimming, beginning of turn, and beginning of strokes preparation detection, respectively. Since the mean and standard deviation of error for detecting these events were higher than others in most cases (e.g., for sacrum and head), part of the error is due to observer error in validation.

In terms of usability, sacrum, head, and right wrist are suitable locations, as they can easily fit into the swimming suit and goggles or be used as a watch. It is observed that head is capable of macro level analysis with lower standard deviation and higher accuracy than wrist or shank. Other than its performance for *Swim*_*B*_ detection, head seems to be the second promising location for micro analysis. Right wrist or right shank both suffer from high error in both macro and micro levels, which might be the result of intra-swimmer variability. As a biomechanically driven approach, macro-micro analysis can provide a detailed view about the nature of movements but its downside is being prone to error caused by technique diversity or being more sensitive to thresholds. Wrist and shank did not perform well with our algorithms and they need further investigation for dealing with their pattern variability.

We included both male and female swimmers, as there were no significant difference between them in the results. Comparing the swimmers due to their individual differences is out of the scope of this study. Since the measurements started from in-water situation, the algorithms cannot cover the dive at the beginning but it is possible to add to our method. The main influence is replacing the wall push-off phase with dive phase. Since we included a moderate pace in our measurements (80% of the best speed), the algorithms are not generalizable to all competitive paces and are valid only within the range of paces included in the measurements. However, improving technique at a moderate pace and then increasing speed is used in training. The use of the highest speed during training is generally required as competitions approach. Therefore, our system can be used in most training sessions where the pace is moderate. Although the validity of our system is not demonstrated by the highest pace, it nevertheless covers a wide range of paces for main swimming techniques. Since we included a moderate pace in our measurements (80% of the best speed), the algorithms are not generalizable to all competitive paces and are valid only within the range of paces included in the measurements. Another limit of this study is the observer error while using the validation system (cameras), showing itself in lap segmentation into swimming phases. Moreover, using camera from the side view, the detection of some events is difficult to observe such as swimming phase start during breaststroke or butterfly, as they are easier to detect in front view.

## Conclusion

The analysis approach proposed in this research detected key temporal events during a training session. It started with finding swimming bouts and laps during a training session. Swimming technique in each lap is then identified, which is useful for finding the lap components during micro analysis. Then each lap is divided into five phases of wall push-off, glide, strokes preparation, swimming, and turn for all techniques. This study has shown that the macro-micro approach with the developed algorithms can cover all the motion phases during a training session. It has been observed that sacrum provides equally good or more promising results than other sensor locations in both levels (other than a few cases such as the beginning of swimming or strokes preparation). In macro level, sacrum achieved the highest accuracy within a range of 0.83–0.98 for swimming bout detection or a range of 0.73–0.97 and 0.82–0.98, respectively, for breaststroke and butterfly technique identification. It also achieved the relatively lower error mean and standard deviation for lap segmentation in most cases. All of these results proves sacrum as the most appropriate sensor location for a single-sensor analysis system, which aims to cover both macro and micro level parameters. To improve the algorithms, we consider investigating machine learning methods, which can better deal with inter and intra variability of swimmers' technique. Future studies with focus on the detailed parameters in each swimming phase will be the next step of the current analysis approach.

## Data Availability Statement

The raw data supporting the conclusions of this article will be made available to qualified researcher, without undue reservation.

## Ethics Statement

This study was approved by EPFL human research ethics committee (HREC, No: 050/2018). Each swimmer participated in the study was informed of the procedure and gave their written consent prior to participation.

## Author Contributions

MH, VG, FD, and KA designed and conceptualized the study, contributed to the analysis, and interpretation of the data. MH carried out the measurements and designed the algorithms. KA supervised the study and FD co-advised it. MH drafted the manuscript. VG, FD, and KA critically revised the manuscript. All authors confirmed the final version and concurred to be responsible for all aspects of this study.

## Conflict of Interest

FD was employed by the company Gait Up. The remaining authors declare that the research was conducted in the absence of any commercial or financial relationships that could be construed as a potential conflict of interest.

## References

[B1] Aung M. S. H. Thies S. B. Kenney L. P. J. Howard D. Selles R. W. Findlow A. H.. (2013). Automated detection of instantaneous gait events using time frequency analysis and manifold embedding. IEEE Trans. Neural Syst. Rehabil. Eng. 21, 908–916. 10.1109/TNSRE.2013.2239313 23322764

[B2] Bächlin M. Förster K. Schumm J. Breu D. Germann J. Tröster G. (2008). “An automatic parameter extraction method for the 7 × 50m stroke efficiency test,” in 2008 Third International Conference on Pervasive Computing and Applications (Alexandria: IEEE), 442–447.

[B3] Barbosa T. M. Keskinen K. L. Fernandes R. Colaço P. Lima A. B. Vilas-Boas J. P. (2005). Energy cost and intracyclic variation of the velocity of the centre of mass in butterfly stroke. Eur. J. Appl. Physiol. 93, 519–523. 10.1007/s00421-004-1251-x 15605282

[B4] Beanland E. Main L. C. Aisbett B. Gastin P. Netto K. (2014). Validation of GPS and accelerometer technology in swimming. J. Sci. Med. Sport 17, 234–238. 10.1016/j.jsams.2013.04.007 23707140

[B5] Bompa T. O. Buzzichelli C. A. (2018). Periodization: Theory and Methodology of Training. 6th ed. Stanningley: Human Kinetics.

[B6] Callaway A. J. Cobb J. E. Jones I. (2010). A comparison of video and accelerometer based approaches applied to performance monitoring in swimming. Int. J. Sports Sci. Coach. 4, 139–153. 10.1260/1747-9541.4.1.139

[B7] Chardonnens J. Favre J. Le Callennec B. Cuendet F. Gremion G. Aminian K. (2012). Automatic measurement of key ski jumping phases and temporal events with a wearable system. J. Sports Sci. 30, 53–61. 10.1080/02640414.2011.624538 22168430

[B8] Cronin J. Rumpf M. (2014). Effect of four different step detection thresholds on nonmotorized treadmill sprint measurement. J. Strength Cond. Res. 28, 2996–3000. 10.1519/JSC.0000000000000497 25250860

[B9] Dadashi F. Aminian K. Crettenand F. Millet G. P. (2013a). “Towards estimation of front-crawl energy expenditure using the wearable aquatic movement analysis system (WAMAS),” in 2013 IEEE International Conference on Body Sensor Networks (Cambridge: IEEE).

[B10] Dadashi F. Crettenand F. Millet G. P. Seifert L. Komar J. Aminian K. (2013b). Automatic front-crawl temporal phase detection using adaptive filtering of inertial signals. J. Sports Sci. 31, 1251–1260. 10.1080/02640414.2013.778420 23560703

[B11] Dadashi F. Millet G. P. Aminian K. (2014). Estimation of front-crawl energy expenditure using wearable inertial measurement units. IEEE Sens. J. 14, 1020–1027. 10.1109/JSEN.2013.2292585

[B12] Dadashi F. Millet G. P. Aminian K. (2015). A Bayesian approach for pervasive estimation of breaststroke velocity using a wearable IMU. Pervasive Mob. Comput. 19, 37–46. 10.1016/j.pmcj.2014.03.001

[B13] Davey N. Anderson M. James D. A. (2008). Validation trial of an accelerometer-based sensor platform for swimming. Sport. Technol. 1, 202–207. 10.1080/19346182.2008.9648474

[B14] Figueiredo P. Barbosa T. M. Vilas-Boas J. P. Fernandes R. J. (2012). Energy cost and body centre of mass' 3D intracycle velocity variation in swimming. Eur. J. Appl. Physiol. 112, 3319–3326. 10.1007/s00421-011-2284-6 22262010

[B15] Figueiredo P. Pendergast D. R. Vilas-Boas J. P. Fernandes R. J. (2013). Interplay of biomechanical, energetic, coordinative, and muscular factors in a 200 m front crawl swim. Biomed. Res. Int. 2013:897232. 10.1155/2013/897232 23586063 PMC3613086

[B16] Fulton S. K. Pyne D. B. Burkett B. (2009). Validity and reliability of kick count and rate in freestyle using inertial sensor technology. J. Sports Sci. 27, 1051–1058. 10.1080/02640410902998247 19642049

[B17] Ge H. Chen G. Yu H. Chen H. An F. (2018). Theoretical analysis of empirical mode decomposition. Symmetry 10:623 10.3390/sym10110623

[B18] Guignard B. Rouard A. Chollet D. Seifert L. (2017). Behavioral dynamics in swimming: the appropriate use of inertial measurement units. Front. Psychol. 8:383. 10.3389/fpsyg.2017.00383 28352243 PMC5348530

[B19] Hagem R. M. O'Keefe S. G. Fickenscher T. Thiel D. V. (2013). Self contained adaptable optical wireless communications system for stroke rate during swimming. IEEE Sens. J. 13, 3144–3151. 10.1109/JSEN.2013.2262933

[B20] Jensen U. Prade F. Eskofier B. M. (2013). “Classification of kinematic swimming data with emphasis on resource consumption,” in 2013 IEEE International Conference on Body Sensor Networks (Cambridge: IEEE).

[B21] Jollife I. T. Cadima J. (2016). Principal component analysis: a review and recent developments. Philos. Trans. R. Soc. A Math. Phys. Eng. Sci. 374:20150202. 10.1098/rsta.2015.0202 26953178 PMC4792409

[B22] Lavoie J. M. Montpetit R. R. (1986). Applied physiology of swimming. Sport. Med. 3, 165–189. 10.2165/00007256-198603030-00002 3520747

[B23] Le Sage T. Bindel A. Conway P. Justham L. Slawson S. West A. (2010). Development of a real time system for monitoring of swimming performance. Proc. Eng. 2, 2707–2712. 10.1016/j.proeng.2010.04.055

[B24] Le Sage T. Bindel A. Conway P. P. Justham L. M. Slawson S. E. West A. A. (2011). Embedded programming and real-time signal processing of swimming strokes. Sport. Eng. 14, 1–14. 10.1007/s12283-011-0070-7

[B25] Lord S. Galna B. Rochester L. (2013). Moving forward on gait measurement: toward a more refined approach. Mov. Disord. 28, 1534–1543. 10.1002/mds.25545 24132841

[B26] Magalhaes F. A. de Vannozzi G. Gatta G. Fantozzi S. (2015). Wearable inertial sensors in swimming motion analysis: a systematic review. J. Sports Sci. 33, 732–745. 10.1080/02640414.2014.962574 25356682

[B27] Marinho D. A. Barbosa T. M. Lopes V. P. Forte P. Toubekis A. G. Morais J. E. (2020). The influence of the coaches' demographics on young swimmers' performance and technical determinants. Front. Psychol. 11:1968. 10.3389/fpsyg.2020.01968 32849152 PMC7431461

[B28] Martens J. Daly D. Deschamps K. Staes F. Fernandes R. J. (2016). Inter-individual variability and pattern recognition of surface electromyography in front crawl swimming. J. Electromyogr. Kinesiol. 31, 14–21. 10.1016/j.jelekin.2016.08.016 27623024

[B29] Mooney R. Corley G. Godfrey A. Osborough C. Newell J. Quinlan L. R.. (2016a). Analysis of swimming performance: perceptions and practices of US-based swimming coaches. J. Sports Sci. 34, 997–1005. 10.1080/02640414.2015.1085074 26359951

[B30] Mooney R. Corley G. Godfrey A. Osborough C. Quinlan L. R. ÓLaighin G. (2015). Application of video-based methods for competitive swimming analysis: a systematic review. Sport. Exerc. Med. Open J. 1, 133–150. 10.17140/SEMOJ-1-121

[B31] Mooney R. Corley G. Godfrey A. Quinlan L. ÓLaighin G. (2016b). Inertial sensor technology for elite swimming performance analysis: a systematic review. Sensors 16:18. 10.3390/s16010018 26712760 PMC4732051

[B32] Morais J. E. Forte P. Nevill A. M. Barbosa T. M. Marinho D. A. (2020). Upper-limb kinematics and kinetics imbalances in the determinants of front-crawl swimming at maximal speed in young international level swimmers. Sci. Rep. 10:11683. 10.1038/s41598-020-68581-3 32669605 PMC7363921

[B33] Morais J. E. Jesus S. Lopes V. Garrido N. Silva A. Marinho D.. (2012). Linking selected kinematic, anthropometric and hydrodynamic variables to young swimmer performance. Pediatr. Exerc. Sci. 24, 649–664. 10.1123/pes.24.4.649 23196769

[B34] Nicol E. Ball K. Tor E. (2018). The characteristics of an elite swimming turn. ISBS Proceedings Archive 36:869.

[B35] Ohgi Y. Ichikawa H. Homma M. Miyaji C. (2003). Stroke phase discrimination in breaststroke swimming using a tri-axial acceleration sensor device. Sport. Eng. 6, 113–123. 10.1007/BF02903532

[B36] Omae Y. Kon Y. Kobayashi M. Sakai K. Shionoya A. Takahashi H. (2017). Swimming style classification based on ensemble learning and adaptive feature value by using inertial measurement unit. J. Adv. Comput. Intell. Intell. Informatics 21, 616–631. 10.20965/jaciii.2017.p0616

[B37] Osborough C. D. Payton C. J. Daly D. J. (2010). Influence of swimming speed on inter-arm coordination in competitive unilateral arm amputee front crawl swimmers. Hum. Mov. Sci. 29, 921–931. 10.1016/j.humov.2010.05.009 20800914

[B38] Pan M. S. Huang K. C. Lu T. H. Lin Z. Y. (2016). Using accelerometer for counting and identifying swimming strokes. Pervasive Mob. Comput. 31, 37–49. 10.1016/j.pmcj.2016.01.011

[B39] Pansiot J. Lo B. Yang G. Z. (2010). “Swimming stroke kinematic analysis with BSN,” in 2010 International Conference on Body Sensor Networks (Singapore: IEEE), 153–158.

[B40] Payton C. J. Bartlett R. M. (1995). Estimating propulsive forces in swimming from three-dimensional kinematic data. J. Sports Sci. 13, 447–454. 10.1080/02640419508732261 8850570

[B41] Pendergast D. R. Di Prampero P. E. Craig A. B. (1980). “Metabolic adaptations to swimming,” in Exercise Bioenergetics and Gas Exchange, eds P. Cerreteli and B. J. Whipp (Amsterdam), 323–336.

[B42] Pereira S. M. Ruschel C. Hubert M. Machado L. Roesler H. Fernandes R. J.. (2015). Kinematic, kinetic and emg analysis of four front crawl flip turn techniques. J. Sports Sci. 33, 2006–2015. 10.1080/02640414.2015.1026374 25813081

[B43] Schmidt R. Lee T. (2019). Motor Learning and Performance: From Principles to Application. 6th ed. Champaign, IL: Human Kinetics Publishers.

[B44] Seifert L. Chollet D. Mujika I. (2011a). World Book of Swimming : From Science to Performance. New York, NY: Nova Science Publishers.

[B45] Seifert L. Leblanc H. Herault R. Komar J. Button C. Chollet D. (2011b). Inter-individual variability in the upper-lower limb breaststroke coordination. Hum. Mov. Sci. 30, 550–565. 10.1016/j.humov.2010.12.003 21439666

[B46] Seifert L. L'Hermette M. Komar J. Orth D. Mell F. Merriaux P. (2014). Pattern recognition in cyclic and discrete skills performance from inertial measurement units. Proc. Eng. 72, 196–201. 10.1016/j.proeng.2014.06.033

[B47] Seifert L. Schnitzler C. Bideault G. Alberty M. Chollet D. Toussaint H. M. (2015). Relationships between coordination, active drag and propelling efficiency in crawl. Hum. Mov. Sci. 39, 55–64. 10.1016/j.humov.2014.10.009 25461433

[B48] Siirtola P. Laurinen P. Roning J. Kinnunen H. (2011). “Efficient accelerometer-based swimming exercise tracking,” in 2011 IEEE Symposium on Computational Intelligence and Data Mining (CIDM) (Paris: IEEE), 156–161.

[B49] Silva A. F. Sousa M. Willig R. Sampaio A. R. Vilas-Boas J. Figueiredo P. (2015). Relationship between strength, stroke efficiency and front crawl swimming performance. Motricidade 15:118.

[B50] Silveira G. A. Araujo L. G. Freitas E. D. S. Schütz G. R. de Souza T. G. Pereira S. M. (2011). Proposal for standardization of the distance for analysis of freestyle flip-turn performance. *Braz. J. Kinanthropometry Hum*. Perform. 13, 177–182. 10.5007/1980-0037.2011v13n3p177

[B51] Slawson S. Conway P. Justham L. Le Sage T. West A. (2010). Dynamic signature for tumble turn performance in swimming. Proc. Eng. 2, 3391–3396. 10.1016/j.proeng.2010.04.163

[B52] Slawson S. E. Justham L. M. Conway P. P. Le-Sage T. West A. A. (2012). Characterizing the swimming tumble turn using acceleration data. Proc. Inst. Mech. Eng. P J. Sport. Eng. Technol. 226, 3–15. 10.1177/1754337111428395

[B53] Stamm A. (2013). Velocity and arm symmetry investigations in freestyle swimming using accelerometry : data collection, analysis and feature extraction (Ph.D. Doctorate). Griffith School of Engineering, Queensland, Australia.

[B54] Stamm A. James D. A. Burkett B. B. Hagem R. M. Thiel D. V. (2013a). Determining maximum push-off velocity in swimming using accelerometers. Proc. Eng. 60, 201–207. 10.1016/j.proeng.2013.07.067

[B55] Stamm A. James D. A. Thiel D. V. (2013b). Velocity profiling using inertial sensors for freestyle swimming. Sport. Eng. 16, 1–11. 10.1007/s12283-012-0107-6

[B56] Vannozzi G. Donati M. Gatta G. Cappozzo A. (2010). “Analysis of swim turning, underwater gliding and stroke resumption phases in top division swimmers using a wearable inertial sensor device,” in Biomechanics and Medicine in Swimming XI, eds P. L. Kjendlie, R. K. Stallman, and J. Cabri (Oslo: Norwegian School of Sport Sciences), 178–180.

[B57] Vantorre J. Chollet D. Seifert L. (2014). Biomechanical analysis of the swim-start: a review. J. Sport. Sci. Med. 13, 223–231. 24790473 PMC3990873

[B58] Wang J. Wang Z. Gao F. Guo M. (2016). SwimSense: monitoring swimming motion using body sensor networks. Lect. Notes Comput. Sci. 9864, 45–55. 10.1007/978-3-319-45940-0_5

[B59] Wright B. V. Stager J. M. (2013). Quantifying competitive swim training using accelerometer-based activity monitors. Sport. Eng. 16, 155–164. 10.1007/s12283-013-0123-1

[B60] Zamparo P. Bonifazi M. Faina M. Milan A. Sardella F. Schena F.. (2005). Energy cost of swimming of elite long-distance swimmers. Eur. J. Appl. Physiol. 94, 697–704. 10.1007/s00421-005-1337-0 15887025

