# Supplementary material for: A Novel Macro-Micro Approach for Swimming Analysis in Main Swimming Techniques Using IMU Sensors
Source: Front Bioeng Biotechnol. 2021 Jan 14;8:597738. doi: 10.3389/fbioe.2020.597738 (PMC7841373; doi:10.3389/fbioe.2020.597738)
Supplement: Supplementary file 1 [file Data_Sheet_1.PDF]

Supplementary Material

1 Rules of algorithms

Table A1 and A2 represent a summary of rules developed for macro and micro swimming analysis and related biomechanics hypothesis. Refer to section 2.1 in the main text for definition of anatomical axes.

Table A1 – Table of rules for macro analysis. The table includes the hypothesis, rule, involved signals and functions for each algorithm developed to find macro parameters on all sensor locations.

**Macro Analysis**				
**Parameter**	**Location**	**Hypothesis**	**Signal(s)**	**Rule**
**Swimming bouts**<br><br>Approximate start and end of swimming, consisting of one or more laps.	SA	The change in trunk posture between standing and prone (for front crawl, butterfly and breaststroke techniques) or supine (for backstroke technique) posture at the beginning and end of each swimming bout will affect sacrum acceleration in sagittal plane.	Acc_y	The derivative of the low pass filtered Acc_y (f_c = 0.1Hz) shows obvious peaks or troughs at the beginning and end of each bout (trough for start and peak for end) because of inclination changes (detectable by a threshold (TH_B)).
	HE	The head posture changes along with trunk between upright and prone or supine posture during each swimming bout start and end which is observable on Acc_y.	Acc_y	The derivative of the low pass filtered Acc_y (f_c = 0.1Hz) shows obvious peaks or troughs at the beginning and end of each bout (trough for start and peak for end) because of inclination changes (detectable by threshold (TH_B)).
	RW	High variability of wrist motion during upper limbs cycles makes this period significant in a full training session. Beginning of a swimming bout is not observable on wrist because no special change was observed before starting the upper limbs cycles.	$\lvert Acc \rvert$	The envelope of $\lvert Acc \rvert$ increases with the start of upper limbs cycles and stays high until the end of swimming bout, which is detectable using an empirical threshold (TH_{BW}).
	RS	Swimmers bend their knee and change their shank inclination for wall push-off before start or at the end of a	Acc_y	The derivative of the low pass filtered Acc_y (f_c = 0.1Hz) shows obvious peaks or troughs (detectable by threshold (TH_B)) because of

		swimming bout, which is observable on Acc_y.		inclination changes (troughs for start and peaks for end).
**Laps Detection**<br><br>Approximate turn identification during each swimming bout	SA	Sudden change in moving direction during simple or tumble turn affects sacrum acceleration. This change happens in all axes but more obviously on Acc_x and Acc_y. However, Acc_x is better than Acc_y because it has a relatively lower amplitude during swimming bout, while it shows the same change in turns	Acc_x	The highest peak of Acc_x during a full swimming bout represent turns (simple or tumble turn).
	HE	Tumble turn and simple turn have different effects on head but during both, there is a quick head motion in one direction (downward for tumble turn (in sagittal plane) and sideways (z axis) for simple turn).	$\lvert Acc_{y,z} \rvert$	The highest peak that exists in $\lvert Acc_{y,z} \rvert$ during a full swimming bout representing the turn (simple or tumble turn).
	RW	During turns, wrist motion has lower magnitude than other part of a swimming bout. Using this decrease in angular velocity level is the key to detecting turns on wrist.	$\lvert Gyr \rvert$	The low pass filtered (f_c = 3Hz) moving average (window size: 1s) of $\lvert Gyr \rvert$ is used for finding a period with relatively lower level of angular velocity. Detection is done by locating approximate turn with a threshold (TH_{LW}).
	RS	During simple turn, shanks move sideways (z axis) quickly to start pushing the wall, while during tumble turn, shanks rotate along with the whole body (in sagittal plane).	Acc_z<br><br>Gyr_z	Relatively large peaks (detectable by threshold (TH_{LS})) appear on Acc_z during simple turn and on Gyr_z during tumble turn.
**Swimming Technique Identification**<br><br>The swimming technique is possible to detect in each lap	SA	In four swimming techniques, sacrum motion is different in terms of angular velocity, gravity direction and motion patterns. Angular velocity is mainly around y axis for front crawl and backstroke, while it is around z axis for breaststroke and butterfly. Supine posture of swimmer during backstroke causes gravity to be in the	Acc_x<br><br>$Gyr_{x,y,z}$	A two-upper-limb-cycle period is located by peak detection on Acc_x in each lap. Front crawl/backstroke category separates from butterfly/breaststroke category using the axis with maximum value of PCA analysis of sacrum $Gyr_{x,y,z}$. In front crawl, the trunk is downward while in backstroke, the trunk is upward (the sign of Acc_x mean is positive for backstroke). Butterfly and breaststroke are

		opposite direction compared to other techniques. Sacrum motion is different in breaststroke and butterfly in terms of motion frequency.		different in terms of dominant frequency of Acc_x, detected with a threshold ($TH_{StyleSA}$).
	HE	Head motion is different in terms of angular velocity, gravity effect and motion patterns. Angular velocity is mainly around y axis for front crawl and backstroke, while it is around z axis for breaststroke and butterfly. Supine posture of swimmer during backstroke causes gravity to have opposite effect compared to other techniques. Head motion is different in breaststroke and butterfly in terms of motion frequency.	$\lvert Acc_{x,y}\rvert$ $Gyr_{x,y,z}$	A two-upper-limb-cycle period is located by peak detection on $\lvert Acc_{x,y}\rvert$ in each lap. Front crawl and backstroke are different in terms of gravity effect on $\lvert Acc_{x,y}\rvert$ (the sign of $\lvert Acc_{x,y}\rvert$ mean is positive for backstroke). Front crawl/backstroke techniques separates from butterfly/breaststroke techniques using PCA analysis of head $Gyr_{x,y,z}$. Butterfly/breaststroke are different in terms of dominant frequency of $\lvert Acc_{x,y}\rvert$ detectable with a threshold ($TH_{StyleHE}$).
	RW	Wrists motion depends on swimmers' learning and technique. The principal component of acceleration shows its highest value on x axis for backstroke technique. Average of acceleration norm is used to identify butterfly since hands motion has the highest average acceleration in butterfly. Between breaststroke and front crawl, the variation of acceleration norm is higher for front crawl.	$Acc_{x,y,z}$ $\lvert Acc\rvert$	A two-upper-limb-cycle period is located by peak detection on $\lvert Acc\rvert$ in each lap. During backstroke, the principal component of acceleration is in x direction. The mean and variation of $\lvert Acc\rvert$ is higher that a threshold ($TH_{StyleWmean}$) for butterfly and front crawl ($TH_{StyleWvar}$) respectively.
	RS	From technique to technique, shank motion is different in terms of principal component of angular velocity and gravity effect. Similar to head and sacrum, gravity effect on shank during backstroke is in opposite compared to other techniques. Breaststroke is the only technique, in which shank motion goes out of sagittal plane. Symmetrical kicks during butterfly is the last clue for separating this technique from front crawl	Acc_x $Gyr_{x,y,z}$	During a five-kick period, gravity effect on Acc_x separated backstroke from other techniques (the sign of Acc_x mean is positive for backstroke). Using a PCA analysis, breaststroke shows its minimum component in y direction because the motion is mainly in shank transverse plane. During butterfly, second component of shank principal angular velocity is positive for right shank (or negative for left shank) due to outward motion of shanks, while it does not necessarily happen for front crawl.

		because it causes a specific rotation in shanks during butterfly.		

Table A2 – Table of rules for micro analysis. The table includes the hypothesis, rule, involved signals and functions for each algorithm developed to find micro parameters on all sensor locations.

Micro Analysis				
**Parameter**	**Location**	**Hypothesis**	**Signal**	**Rule**
**Wall push-off start ($Push_B$)**<br><br>Detected as the frame that trunk starts its forward motion	SA	Sacrum has a motion with high forward acceleration (y axis) at the beginning of wall push-off. This motion leaves a peak in Acc_y, After which the acceleration decreases due to water drag.	Acc_y	Backstroke: The maximum of Acc_y in a 2-second window after swimming bout start is the closest point to push-off start. The result is not sensitive to the length of this window.<br><br>Other: it is easier to find the trough after push-off start and use it to detect the push-off start. In a 2-second window after swimming bout start, Acc_y concavity changes which is detectable on its derivative. If this sample is followed by a trough lower than zero on Acc_y, then the peak before this trough is the closest point to push-off start. The result is not sensitive to the length of the window.
	HE	Head has a motion with high forward acceleration (y axis) at the beginning of wall push-off. This motion leaves a peak in Acc_y and $\lvert Acc \rvert$, After which the acceleration decreases due to water drag.	Acc_y<br><br>$\lvert Acc \rvert$	Backstroke: The maximum of Acc_y in a 2-second window after swimming bout start is the closest point to push-off start. The result is not sensitive to the length of the window.<br><br>Other: in a 2-second window after swimming bout start, the maximum of $\lvert Acc \rvert$ provides a rough approximation which will be finetuned with a close peak on Acc_y. The result is not sensitive to the length of the window.
	RW	The swimmer wrists goes down in water for push-off, which causes an acceleration against gravity on axis y. As the swimmer is raising and stretching the arms forward, this acceleration increases.	Acc_y	As Acc_y decreases below zero due to wrist downward motion, it increases afterwards when the swimmer raises their wrists for push-off. The trough before this increase in Acc_y is detected as the closest to push-off start.
	RS	Swimmer's knees extend during push-off after an almost motionless period, so this sharp increase in	$\lvert Gyr \rvert$	As knees starts to extend at the beginning of push-off, $\lvert Gyr \rvert$ increases and shows a peak.

		$\lvert Gyr \rvert$ is a sign of push-off start.		The first minimum before this peak is the closest point to push-off start.
**Glide start ($Glid_B$)**<br><br>Detected as the frame that swimmer's feet leave the wall	SA	Sacrum forward acceleration (y axis) gets closer and closer to zero due to the end of push-off period and deceleration start.	Acc_y	The first negative trough on Acc_y after push-off start shows the beginning of glide period that the swimmer's body starts to decelerate.
	HE	Head forward acceleration (y axis) should get close to zero due to the end of push-off period.	Acc_y	The first negative trough on Acc_y after push-off start shows the beginning of glide period that the swimmer's body decelerates.
	RW	As the arms are fully stretched, Acc_y at glide start will be a sample closest to zero after push-off start.	Acc_y	As push-off start on wrist was a negative trough, after which the acceleration started increasing towards zero, the first peak on Acc_y after push-off start is the closest to glide start. It is close to zero.
	RS	$\lvert Gyr \rvert$ of Shank decreases close to zero due to the end of push-off period.	$\lvert Gyr \rvert$	The first trough of $\lvert Gyr \rvert$ after push-off start, where angular velocity is almost zero.
**Strokes Preparation start ($StPr_B$)**<br><br>Detected as the frame that feet start the dolphin kick or kick and hand in breaststroke	SA	The wave generated in swimmer's body due to strokes preparation kicks causes a periodic change in sacrum acceleration after a motionless glide. The change is clear on sacrum Acc_x because of its upward and downward motion.	$\lvert Acc_x \rvert$	Start of this phase is detectable by using two thresholds on peaks of $\lvert Acc_x \rvert$ (TH_{SPSA}) and its variation ($TH_{SPSAvar}$) obtained with moving standard deviation (100 samples window size). As soon as they get higher than the thresholds, strokes preparation phase is started. The first peak or trough (the ones happening earlier) of this period is considered as strokes preparation start.
	HE	The thrust caused by strokes preparation kicks will show a periodic change on head Acc_y. This change happens after the motionless period of glide.	$\lvert Acc_y \rvert$	The first peak of this periodic change in $\lvert Acc_y \rvert$ is considered as strokes preparation start. It is detected using thresholds on peak magnitude (TH_{SPHE}) and prominence ($TH_{SPHEprom}$).
	RW	As wrist is almost motionless before strokes preparation start, its $\lvert Acc \rvert$ remains equal to g. Strokes preparation kicks causes a wave motion on wrists too.	$\lvert Acc \rvert$	The first peak on $\lvert Acc \rvert$, detected by a threshold (TH_{SPRW}) after motionless glide phase is considered as strokes preparation start.

	RS	Upward and downward motion of shanks during the dolphin kicks after a motionless period is the clue to find stroke preparation start. It is observable on all axes of shank acceleration.	Acc_y<br>$\|Acc_x\|$<br>$\|Acc\|$	Backstroke: The first positive peak on Acc_y after glide start caused by kicking thrust is the start of strokes preparation phase.<br><br>Other: The first peak on $\|Acc_x\|$ bigger than a threshold (TH_{SPRS}) is close to the answer. The first sample before this peak where $\|Acc\|$ passes g is the start of strokes preparation phase because the shanks are motionless and $\|Acc\| = g$ before strokes preparation start
**Swimming start ($Swim_B$)**<br><br>Detected as the frame of first upper limbs cycle	SA	Sacrum motion changes with the start of upper limbs cycles. For front crawl and backstroke, the sacrum angular velocity is in sagittal plane (XY plane) during strokes preparation phase while it changes to frontal plane (XZ plane) during upper limbs cycles. For breaststroke and butterfly the motion changes in terms of energy increment of Gyr_z and Acc_y with start of swimming phase.	Gyr_y<br>$\|Gyr_y\|$<br>Gyr_z<br>Acc_y	Front crawl & backstroke: Gyr_y becomes prominent after the first upper limbs cycle start because of body rolling motion. It is detected with a threshold ($TH_{SSA-FCBaS}$) on $\|Gyr_y\|$ and then finetuned with the closest peak of Gyr_y before this sample.<br><br>Breaststroke: Using Empirical Mode Decomposition (EMD) and Hilbert-Huang transform, abrupt increase in instantaneous energy level of second mode of filtered Gyr_z (f_c = 2 Hz) happens in swimming phase. The sample is detected using a threshold ($TH_{SSA-BrS}$) and the previous peak on Gyr_z is close to swimming phase start.<br><br>Butterfly: Acc_y is decomposed into several components using EMD method. The second component starts a periodic change after swimming phase start and the first peak on it is detected with a threshold (TH_{SSA-BF}). The trough before this peak is close to swimming phase start.
	HE	Head motion changes due to swimming phase start varies from technique to technique. In front crawl, head starts to roll, causing an increase in Gyr_y energy level. For butterfly and breaststroke, head upward and downward motion intensifies, which causes an increase in Gyr_z energy level. In backstroke, head become steadier after upper limbs cycles start, which means less energy level of Gyr_z .	Gyr_y<br>Gyr_z	Front crawl: Threshold-based detection (TH_{SHE-FC}) of Gyr_y energy level (obtained with HHT(Gyr_y)) increase is used to find the vicinity of upper limbs cycle start. The first trough before this increment is chosen as swimming start.<br><br>Butterfly & breaststroke: Threshold-based detection ($TH_{SHE-BFBrS}$) of Gyr_z energy level increase (obtained with HHT(Gyr_z)) is used to find the vicinity of swimming start. The first trough before this increment is chosen as swimming start.<br><br>Backstroke: Threshold-based detection ($TH_{SHE-BaS}$) of Gyr_z energy level decrease (obtained with HHT(Gyr_z)) is used to find the vicinity of swimming start. The first peak

				before this decrement is chosen as swimming start.
	R&LW	Wrists start to move at the beginning of swimming phase, which is observable on acceleration and angular velocity. For front crawl and butterfly, Acc_y change is easier to detect because of hands downward motion right from the swimming start. For breaststroke, hand rotation at the swimming phase start causes a change in Gyr_y and Acc_x .	Acc_x<br>Acc_y<br>Gyr_y	For front crawl and backstroke, the algorithm is implemented on both wrists and the earlier result was chosen as the answer.<br><br>Front crawl & butterfly: The trough before the first peak on Acc_y caused by upper limbs cycles after strokes preparation start is swimming start.<br><br>Breaststroke: Gyr_y first peak after strokes preparation start an approximate period for the answer. The trough before the first peak of filtered Acc_x (f_c = 5 Hz) happening in this period is the closest to swimming start.<br><br>Backstroke: The trough before the first peak on Acc_x caused by upper limbs cycles after strokes preparation start is swimming start.
	RS	Start of upper limbs cycles on shanks causes a change in the kicking method depending on swimming technique. Except for breaststroke, kicking happens in sagittal plane and Acc_x is the signal that changes more obviously. The same happens on Acc_y for breaststroke. This change in kicking is possible to detect as the beginning of swimming phase.	Acc_x<br>Acc_y	Using EMD method on Acc_y for breaststroke and Acc_x for the rest techniques, the second mode separates the fluctuations after swimming start on shanks. Threshold-based peak detection (TH_{SRS}) finds the first peak of this mode and the trough before it is considered as swimming start.
**Turn start ($\boldsymbol{Turn_B}$)**<br><br>Detected as the frame that head starts the downward motion / hands touch the wall)	SA	During tumble and simple turn, sacrum motion is mainly in sagittal and frontal plane respectively. In both cases, Acc_x is affected by the motion and shows a sudden change. Approximate turn, which is a sample during turn phase is already detected with lap detection algorithm and is used here.	Acc_x	Backstroke: it happens at a peak caused by the rolling before turn. This peak is the same as approximate turn already detected on Acc_x.<br><br>Other: in a period before approximate turn, the turn causes a large peak in Acc_x, the trough before which is close to turn start.
	HE	For tumble turn, head starts the downward motion, which can be observed as a	$\|Acc\|$	Front crawl & backstroke: $\|Acc\|$ shows a peak which is close to approximate turn. The sample

		big peak on head $\|Acc\|$. Before turning, head rests for a short period where $\|Acc\|$ should be close to g. For simple turn, the motion is basically in frontal plane which is detectable on head Gyr_x.	Gyr_x	before this peak where acceleration is equal to g is close to turn start.<br><br>Butterfly & breaststroke: Gyr_x has a peak close to approximate turn. Turn start is the trough before this peak
	RW	During tumble turn, wrists undergo a complete turn from prone to supine along with the whole body. This change is clear on Acc_y. During simple turn, forearm orientation changes from horizontal before turn to vertical (or close to vertical) and again to horizontal after turn. This change is observable on wrist Acc_y.	Acc_y	Front crawl & backstroke: wrist Acc_y shows a peak close to approximate turn, clear on high-filtered Acc_y (f_c = 2 Hz). The trough before this peak is close to turn start.<br><br>Breaststroke & butterfly: Acc_y shows a peak close to approximate turn. The trough before this peak is close to turn start.
	RS	During tumble turn, shanks rotate with the whole body, causing a clear change on Gyr_z, while during simple turn, they move sideways (z axis) to reach the wall for push-off.	Gyr_z<br><br>Acc_z	Front crawl & backstroke: shank Gyr_z increases abruptly during rotation, close to approximate turn. The trough before this change is turn start.<br><br>Breaststroke & butterfly: Acc_z increases because of the shank sideway motion, close to approximate turn. The trough before this increase is turn start.

2 Sensitivity analysis of thresholds

The thresholds are changed in both directions according to the percent declared and the total change in algorithm result (accuracy and precision of swimming bouts and lap detection and swimming technique identification in macro analysis and the estimated values for phase starts in micro analysis) is reported. The percent of change depends on how the results changed with a least amount of 10%.

Table A3 –Table of thresholds sensitivity analysis

Threshold	Description	Threshold change (%)	Results change (%)
TH_B	The thresholds used for swimming bouts detection on SA, HE and RS	30	0
TH_{BW}	The threshold used for swimming bouts detection on RW	15	5
TH_{LW}	The threshold used for swimming lap detection on RW	30	0
TH_{LS}	The threshold used for swimming lap detection on RS	15	5
$TH_{StyleSA}$	The threshold used for swimming technique identification on SA	30	Less than 1
$TH_{StyleHE}$	The threshold used for swimming technique identification on HE	30	Less than 1
$TH_{StyleWmean}$	The first threshold used for swimming technique identification on W	10	5
$TH_{StyleWvar}$	The second threshold used for swimming technique identification on W	50	1
TH_{SPSA}	The first threshold used for $StPr_B$ detection on SA	This threshold is 1g and can be justified biomechanically	
$TH_{SPSAvar}$	The second threshold used for $StPr_B$ detection on SA	30	0
TH_{SPHE}	The first threshold used for $StPr_B$ detection on HE	20	12
$TH_{SPHEprom}$	The second threshold used for $StPr_B$ detection on HE	10	15
TH_{SPRW}	The first threshold used for $StPr_B$ detection on RW	10	5
TH_{SPRS}	The first threshold used for $StPr_B$ detection on RS	15	5
$TH_{SSA-FCBaS}$	The threshold used for $Swim_B$ detection on SA for front crawl and backstroke	15	5
$TH_{SSA-BrS}$	The threshold used for $Swim_B$ detection on SA for breaststroke	20	Less than 1
TH_{SSA-BF}	The threshold used for $Swim_B$ detection on SA for butterfly	15	Less than 1
TH_{SHE-FC}	The threshold used for $Swim_B$ detection on HE for front crawl	20	Less than 1
$TH_{SHE-BFBrS}$	The threshold used for $Swim_B$ detection on HE for butterfly and breaststroke	10	5
$TH_{SHE-BaS}$	The threshold used for $Swim_B$ detection on HE for backstroke	15	9
TH_{SRS}	The threshold used for $Swim_B$ detection on RS	10	5

3 Glossary of terms

Here if the table of glossary of all the terms used for macro/micro approach of swimming analysis.

Table A4 – Table of glossary

Term	Definition
IMU	Inertial measurement unit
Acc	Acceleration data (g)
Gyr	Gyroscope data ($°/s$)
$\|Acc_{y,z}\|$	Sum of the acceleration on y and z axes
$Gyr_{x,y,z}$	All three axes of gyroscope
$\|Gyr\|$	Norm of angular velocity
$\dot{Acc_y}$	Derivative of Acc_y
SA	Sacrum
HE	Head
RW	Right wrist
RS	Right shank
Swimming bout	The swimming parts (in any swimming technique) during a training session that includes one or more laps. It start with an approximate start of swimming and finishes with an approximate end of it.
Swimming lap	The wall-to-wall period of swimming (in any swimming technique) that starts with wall push-off and ends when the swimmer starts the next wall push-off after turn or touches the wall (at the end of the swimming bout)
Simple turn	The turn at the end a the swimming lap during breaststroke and butterfly swimming techniques
Tumble turn	The turn at the end a the swimming lap during front crawl and back swimming techniques
Swimming technique	The technique of swimming which is one among this list: Front crawl, Breaststroke, Butterfly, Backstroke
Swimming phase	Each swimming lap is divided in five swimming phases (wall push-off, glide, strokes preparation, swimming and turn)
$Push_B$	Beginning of wall push-off phase
$Glid_B$	Beginning of glide phase
$StPr_B$	Beginning of strokes preparation phase
$Swim_B$	Beginning of swimming phase
$Turn_B$	Beginning of turn phase
Δ-	Stands for the duration of each phase
TH_B	The thresholds used for swimming bouts detection on SA, HE and RS
TH_{BW}	The threshold used for swimming bouts detection on RW
TH_{LW}	The threshold used for swimming lap detection on RW
TH_{LS}	The threshold used for swimming lap detection on RS
$TH_{StyleSA}$	The threshold used for swimming technique identification on SA
$TH_{StyleHE}$	The threshold used for swimming technique identification on HE
$TH_{StyleWmean}$	The first threshold used for swimming technique identification on W
$TH_{StyleWvar}$	The second threshold used for swimming technique identification on W
TH_{SPSA}	The first threshold used for $StPr_B$ detection on SA
$TH_{SPSAvar}$	The second threshold used for $StPr_B$ detection on SA
TH_{SPHE}	The first threshold used for $StPr_B$ detection on HE
$TH_{SPHEprom}$	The second threshold used for $StPr_B$ detection on HE
TH_{SPRW}	The first threshold used for $StPr_B$ detection on RW
TH_{SPRS}	The first threshold used for $StPr_B$ detection on RS
$TH_{SSA-FCBaS}$	The threshold used for $Swim_B$ detection on SA for front crawl and backstroke
$TH_{SSA-BrS}$	The threshold used for $Swim_B$ detection on SA for breaststroke
TH_{SSA-BF}	The threshold used for $Swim_B$ detection on SA for butterfly
TH_{SHE-FC}	The threshold used for $Swim_B$ detection on HE for front crawl

$TH_{SHE-BFBrS}$	The threshold used for $Swim_B$detection on HE for butterfly and breaststroke
$TH_{SHE-BaS}$	The threshold used for $Swim_B$detection on HE for backstroke
TH_{SRS}	The threshold used for $Swim_B$detection on RS
